# Development of a urinometer for automatic measurement of urine flow in catheterized patients

**DOI:** 10.1371/journal.pone.0290319

**Published:** 2023-08-31

**Authors:** José-Luis Lafuente, Samuel González, Enrique Puertas, Vicente Gómez-Tello, Eva Avilés, Niza Albo, Claudia Mateo, Juan-Jose Beunza

**Affiliations:** 1 IASalud, Universidad Europea de Madrid, Villaviciosa de Odón, Madrid, Spain; 2 Engineering Department, School of Architecture, Engineering, & Design, Universidad Europea de Madrid, Villaviciosa de Odón, Madrid, Spain; 3 Intensive Care Unit, Hospital Universitario HLA Moncloa, Madrid, Spain; 4 Department of Medicine, Universidad Europea de Madrid, Villaviciosa de Odón, Madrid, Spain; 5 Science, Computing and Technology, School of Engineering, Architecture & Design, Universidad Europea de Madrid, Villaviciosa de Odón, Madrid, Spain; 6 Emergency Department, Hospital Universitario HLA Moncloa, Madrid, Spain; 7 Research and Doctorate School, Universidad Europea de Madrid, Villaviciosa de Odón, Madrid, Spain; Chitkara University, INDIA

## Abstract

Urinary flow measurement and colorimetry are vital medical indicators for critically ill patients in intensive care units. However, there is a clinical need for low-cost, continuous urinary flow monitoring devices that can automatically and in real-time measure urine flow. This need led to the development of a non-invasive device that is easy to use and does not require proprietary disposables. The device operates by detecting urine flow using an infrared barrier that returns an unequivocal pattern, and it is capable of measuring the volume of liquid in real-time, storing the history with a precise date, and returning alarms to detect critical trends. The device also has the ability to detect the color of urine, allowing for extended data and detecting problems in catheterized patients such as hematuria. The device is proposed as an automated clinical decision support system that utilizes the concept of the Internet of Medical Things. It works by using a LoRa communication method with the LoRaWAN protocol to maximize the distance to access points, reducing infrastructure costs in massive deployments. The device can send data wirelessly for remote monitoring and allows for the collection of data on a dashboard in a pseudonymous way. Tests conducted on the device using a gold standard medical grade infusion pump and fluid densities within the 1.005 g/ml to 1.030 g/ml urine density range showed that droplets were satisfactorily captured in the range of flows from less than 1 ml/h to 500 ml/h, which are acceptable ranges for urinary flow. Errors ranged below 15%, when compared to the values obtained by the hospital infusion pump used as gold standard. Such values are clinically adequate to detect changes in diuresis patterns, specially at low urine output ranges, related to renal disfunction. Additionally, tests carried out with different color patterns indicate that it detects different colors of urine with a precision in detecting RGB values <5%. In conclusion, the results suggest that the device can be useful in automatically monitoring diuresis and colorimetry in real-time, which can facilitate the work of nursing and provide automatic decision-making support to intensive care physicians.

## Introduction

Caring for critically ill patients in the Intensive Care Unit (ICU) involves monitoring vital signs in real-time. An automatic early warning system would enable healthcare professionals to address significant variations that could potentially be dangerous [1,2]. One of the ways to assess a patient’s renal function is by measuring urine flow, which serves as a general indicator to determine the degree of vascular perfusion in organs [3]. The most commonly used measure is urine flow, which is calculated as the volume of urine produced per unit of time. By continuously monitoring urine production, healthcare professionals can establish an early alert system for acute renal injury, similar to the canary in a coal mine proverb [4].

The current method for measuring a patient’s urine flow rate in the ICU involves a container with volume markings, which nurses use to manually calculate urine volume once an hour. However, this method is prone to measurement and recording errors, and can result in a delay of one or more hours in detecting pathologies such as oliguria (< 25 mL/h) or anuria (< 15 mL/h) [5]. Additionally, this process requires a significant amount of time—for example, in an ICU with 15 beds, it is estimated that 12 hours a day are spent taking these measurements, assuming one measurement per hour and 2 minutes per patient.

Scientific evidence supports the use of an automatic urinometer that can detect different pathologies through a pattern detection system in critical patients [6–8].

Some theoretical investigations [9–14] and patent publications [15–23] have aided in technological progress; however, these solutions frequently become obsolete and do not provide complete effectiveness. Our research suggests that, at present, no fully matured commercial products fulfill the fundamental criteria for functionality or practical implementation [7,24–34]. The devices in existence depend on costly and exclusive disposable components, constraining their prospects for extensive adoption. Most of the products we have found are discontinued. Table 1 displays the main products identified. Some market products, which do not provide product information, have been excluded.

**Table 1:** List of the main commercial urinometer models.

Model	Maker	Sensor Type	Additional Sensors	Wi-Fi / BLE	Other Functions	Price	Status
**SIPPI**	Observe Medical	Capacitive				Console: €1500 Bag: €20	●
**CLARITY RMS**	Renal Sense	Flow sensor			Bladder temperature	Console: £3810 Sensor: £65	●
**ACCURYN**	Potrero	Photoelectric	Temperature in catheter	●	Bladder temperature	Console: $2124 Catheter: $223	●
**URINFO 2000**	FlowSense	Photoelectric					X
**CRITICORE MONITOR**	Bard Medical	Photoelectric	Temperature in catheter	●	Bladder temperature		X
**RENAL GUARD SYSTEM**	RenalGuard	Weighing			Infusion volume	Console: $800	●
**FOM-200**	QMD	Photoelectric	Pressure		Bladder/abdominal pressure		X
**SENTINEL**	Serenno Medical	Pneumatic		●		Console: $1000–2000 Sensor: $25	●
**AKAS**	Uromon			●			●
**SENSICA**	BD, formerly C.R. Bard, Inc.	Weighing		●			?
**XIAN HUIZHI**		Photoelectric	Weighing and pressure		Bladder pressure		?
**TOP-1000**		Photoelectric		●			?

It is estimated that there are 73,585 ICU beds in Europe [35]. Assuming an occupancy rate between 61.6% (Greece) and 94.9% (Ireland), and a catheterization rate of 76% [36], we estimate that there are between 34,450 and 53,072 catheterized patients admitted to ICUs in Europe at any given static point in time. The value of a low-cost urinometer does not lie in reducing the cost of the current system (manual, labor cost), but in globally replacing it in a scalable way that is economically viable for all healthcare systems, including those in high-income countries.

From now onwards, we will call our prototype UrinAI.

The structure of this manuscript is organized as follows. In the "Materials and Methods" section, we detail the requirements and the prototype design process, encompassing individual components such as the sensor module, data storage, power supply, and communications, among other facets. Subsequently, we introduce the construction of a realistic simulation model. Results derived from tests conducted in the laboratory environment are presented in the "Results" section, followed by a procedure for device utilization. Lastly, the "Discussion and Conclusions" section is elaborated upon.

## Materials and methods

### Prototype requirements

Based on the clinical needs expressed by our doctors and the results of various systematic reviews, we identified five fundamental requirements UrinAI the device should have. First, it should minimize contact with urine. Second, it should be able to detect changes in flow patterns. Third, it should not depend on exclusive disposable parts. Fourth, it should be easy to use by healthcare professionals. Fifth, it must be cost-effective.

To be economically viable, the device’s price must be affordable for all potential clinical users. Additionally, it should save time for health personnel by having these parameters controlled without the need for own disposables. In other words, we aimed for the automation of data collection.

Furthermore, adding the following functionalities would create an optimized device that provides more value without the need to make it expensive or complex. First, a real-time clock (RTC) could allow users to control time more accurately and help record more precise moments of each measurement for in-depth study. Second, it could offer the possibility of extending to new functions or other measurement parameters through the I^2^C BUS, leaving the system open to possible improvements or additions. Third, the device could include colorimetric analysis of urine, which would add value to clinical care [37–40]. Fourth, it could have varied communication systems, such as WIFI, Bluetooth Low Energy (BLE), and LoRa, to choose from according to individual needs. We believe LoRa would be the best choice due to its long-range indoors, low power consumption, and its use of only a few multi-byte packets of information [41–47]. Finally, the device could include a backup MicroSD card for data registration.

### Development of the prototype

After evaluating various alternatives to meet the four fundamental requirements, we decided to use an intravenous infusion dropper system that incorporates an electronic sensor to capture the number of drops and process this information. This method allowed us to use a non-invasive approach, as we didn’t need to touch the urine, and it enabled us to monitor changes in the urine flow. Additionally, the dropper system is disposable, widely available in clinical settings, and familiar to health personnel, making it easy to use.

**Fig 1:**
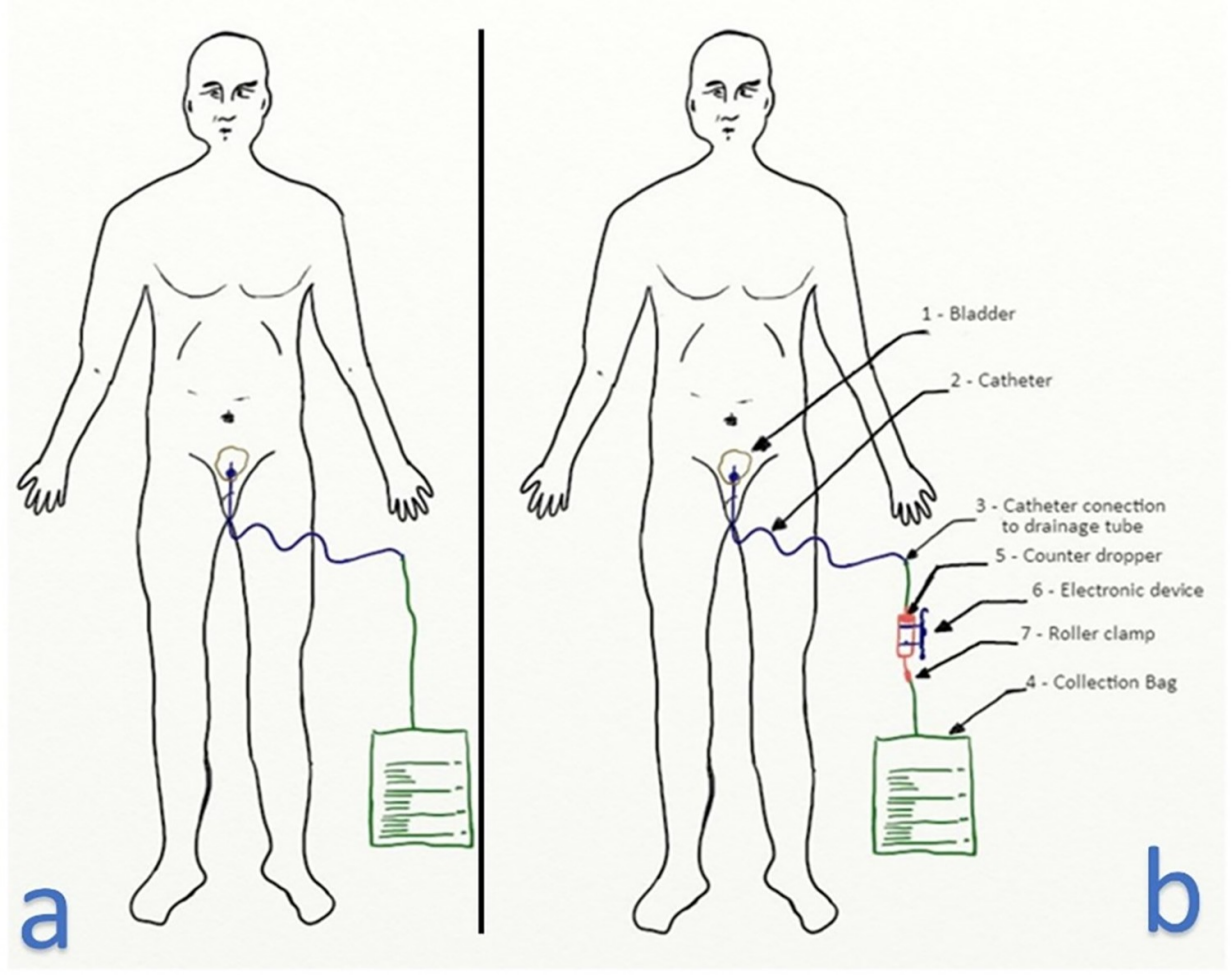
Patient with catheter. (a) Patient probed normally. (b) Patient probed with inline urinometer.

In order to implement our system, we inserted an intravenous infusion dropper between the Foley catheter tube and the urine bag, as shown in Fig 1B. The Foley catheter passed through the patient’s urethra and into the bladder, where it is typically connected to the urine bag. Our electronic device was positioned around the dropper, which is the disposable element, to capture the urine flow without any physical contact. The urinometer we proposed operates on the principle of gravity.

The flow of urine through the ureters and urethra is typically less than the flow admitted by the dropper. Therefore, it is unlikely that the dropper would pose any issue for the normal flow of the patient’s urine, given normal physiological and pathophysiological conditions (excluding rare pathologies of the ureters and/or urethra). Although the flow of urine is dependent on the muscular contraction of the bladder wall, in most cases, when the patient is catheterized, the flow of urine occurs by gravity (which is why the urine bag is placed under the patient’s bed, remaining below the bladder).

However, as the patient moves and the bladder is a bag, an accumulation of fluid can occur in the bladder that can be released instantly, causing a jet flow due to free fall. As the dropper is sterile and the only element in contact with the urine, we can guarantee a minimum risk of retrograde contamination. For more detailed explanations of the physiological aspects refer to S1 Appendix.

#### Sensor module

**Fig 2:**
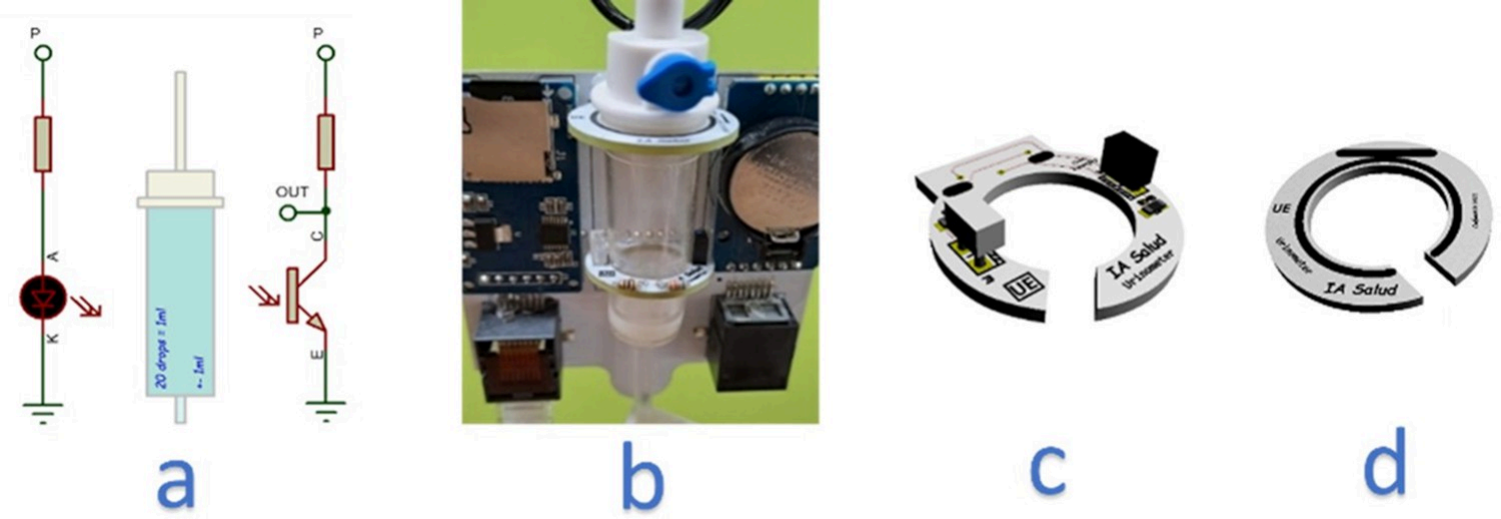
Infrared barrier: Sensor implementation and mounting. (a) Schematic drawing of the infrared barrier. (b) View of the dripper attached to the electronic device using two fixation rings. (c) Implementation of the sensor on PCB, bottom ring. (d) Top Attachment Ring PCB.

The sensor module consists of an infrared barrier designed to capture droplets. This barrier was made up of an infrared beam created by an IR LED and a phototransistor, as illustrated in Fig 2A. This approach was selected as the most suitable solution due to its feasibility, robustness, and cost-effectiveness in meeting the requirements for accurate detection of changes in flow. The infrared barrier was optimized to cover the appropriate field of view (FOV). This type of detection is based on the principle of interrupting an infrared light beam. Both the emitter and receiver need to operate at the same wavelength within the infrared spectrum, specifically at 880 nm in our urinometer. The distance between the emitter and the phototransistor is a critical factor. Maintaining precise alignment and appropriate geometry is essential for optimizing the detection range. Parameters such as wavelength, application voltage, current ranges, viewing angle, and lens geometry are determined by the manufacturer of the infrared emitter and receiver and are provided in the datasheets. The emitter and receiver are paired by the manufacturer to work together. The phototransistor incorporates a UV filter that minimizes the risk of environmental interference, such as direct sunlight or nearby intense light sources. The accurate selection of the emitter-receiver pair, along with adjustable parameters during the hardware design phase, ensured that the infrared light beam operates optimally in our application. Our system is hardware-calibrated to meet the application’s specifications, leaving base level and measurement level calibration of the infrared barrier interruption to the firmware.

For our prototype, we used an infusion dropper with a diameter of 4x3.1 mm, which corresponds to the dropper manufacturer’s formula:

20droplets=1ml±0.1ml (1)

We considered this level of error to be acceptable for our purposes, especially if the error is systematic.

Fig 2B illustrates the positioning of the drop-counter in relation to the electronic device. The dripper was secured using two rings, as depicted in Fig 2C and 2D. These rings allowed for the dripper to be attached securely, with the lower ring featuring an infrared sensor for detecting patterns of drops, as shown in Fig 2C. The upper ring had no associated electronic parts, and both rings had openings to facilitate the placement of the dropper, with the exit line passing through the openings depicted in Fig 2C and 2D.

The specific choice of 25 degrees viewing angle ensured detection within the dropper chamber’s spatial confines. This, in conjunction with the infrared emitter’s intensity and the phototransistor’s sensitivity, guaranteed detection at the distance between the emitter and receiver. In this instance, that distance was the dropper’s external diameter, which was inherently small.

According to Formula (1), only the droplets passing through the infrared barrier should be counted, which we will discuss later. Each droplet formed a unique pattern that we could detect with certainty, allowing us to differentiate and quantify the pattern of droplets or other events, such as a jet. In the case of a jet flow, we could estimate the volume of urine, though this feature is not currently implemented. The barrier also enabled us to determine whether the dripper is present or not, as it emits different voltages depending on whether the infrared barrier was interrupted or not. If the dripper is present, the infrared beam will cross the two translucent walls of the dripper.

We refer to the voltage emitted when the dropper is present and uninterrupted by any droplet as the "baseline voltage". This voltage was self-calibrated and could vary depending on factors such as the opacity of the dropper wall.

To ensure accurate measurement of the droplets crossing the IR barrier, various parameters such as wavelength, FOV, and response times were parameterized. A sender-receiver pair with the same wavelength on an infrared spectrum was selected, with the receptor being immune to ultraviolet and environmental light to prevent interference with the measuring system. These parameters, along with the desired shape of the components, guided the search for the appropriate infrared light emitter diode (LED IR) and infrared phototransistor from commercially available options.

**Fig 3:**
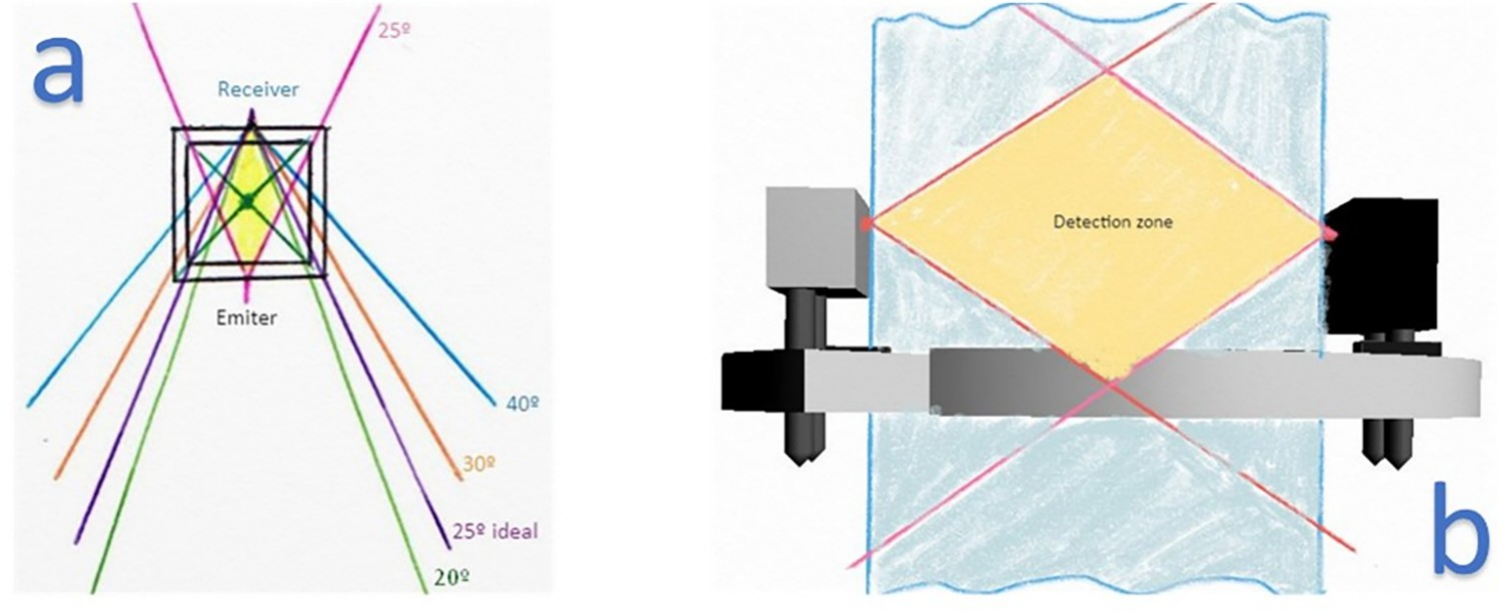
Field of vision study. (a) We selected 25 degrees as the one that best suited our requirement. (b) View with sensor ring and detection zone.

In order to detect droplets within the entire internal space of a dropper, the necessary field of view (FOV) for both the receiver and emitter was calculated. This space is referred to as the "chamber". Once the ideal FOV was determined, a sender-receiver pair with that specific viewing angle was sought. The FOV, along with the wavelength, was determined during the manufacture of the emitters and receivers and couldn’t be adjusted afterwards. For this particular study, an FOV of 25 degrees was deemed appropriate. This allowed the sensor to only detect droplets within the required space inside the dropper, while also providing a tolerance for tilting. Outside of this intersection zone between the IR LED light and the phototransistor, the sensor was not affected by external disturbances (Fig 3).

**Fig 4:**
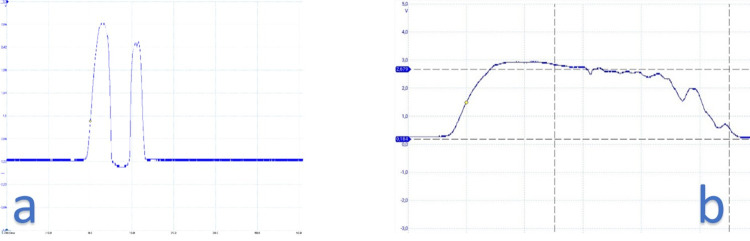
Droplet pattern observed with the oscilloscope. (a) Each drop created a unique pattern. (b) Jet example.

The phototransistor included an ultraviolet light filter to prevent interference from external sources such as sunlight or lighting lamps. This, along with a specific wavelength and droplet pattern sequence, made the sensor resistant to external disturbances. Each droplet passing through the detection zone created a specific wave pattern, which allowed for the identification of different cases. The phototransistor returned a specific waveform for each different case, such as detecting the presence of the dropper, droplets passing through (Fig 4A), or liquid passing through in a jet (Fig 4B).

The shape of each droplet that passed through the infrared beam could be represented by a distinct waveform pattern. This pattern could be detected with high accuracy to avoid any potential errors. Experimental tests have confirmed that all droplets were successfully captured. Consequently, the urine flow rate could be calculated by counting the number of times the barrier was interrupted and applying the appropriate volume assigned to each droplet according to Formula (1).

**Fig 5:**
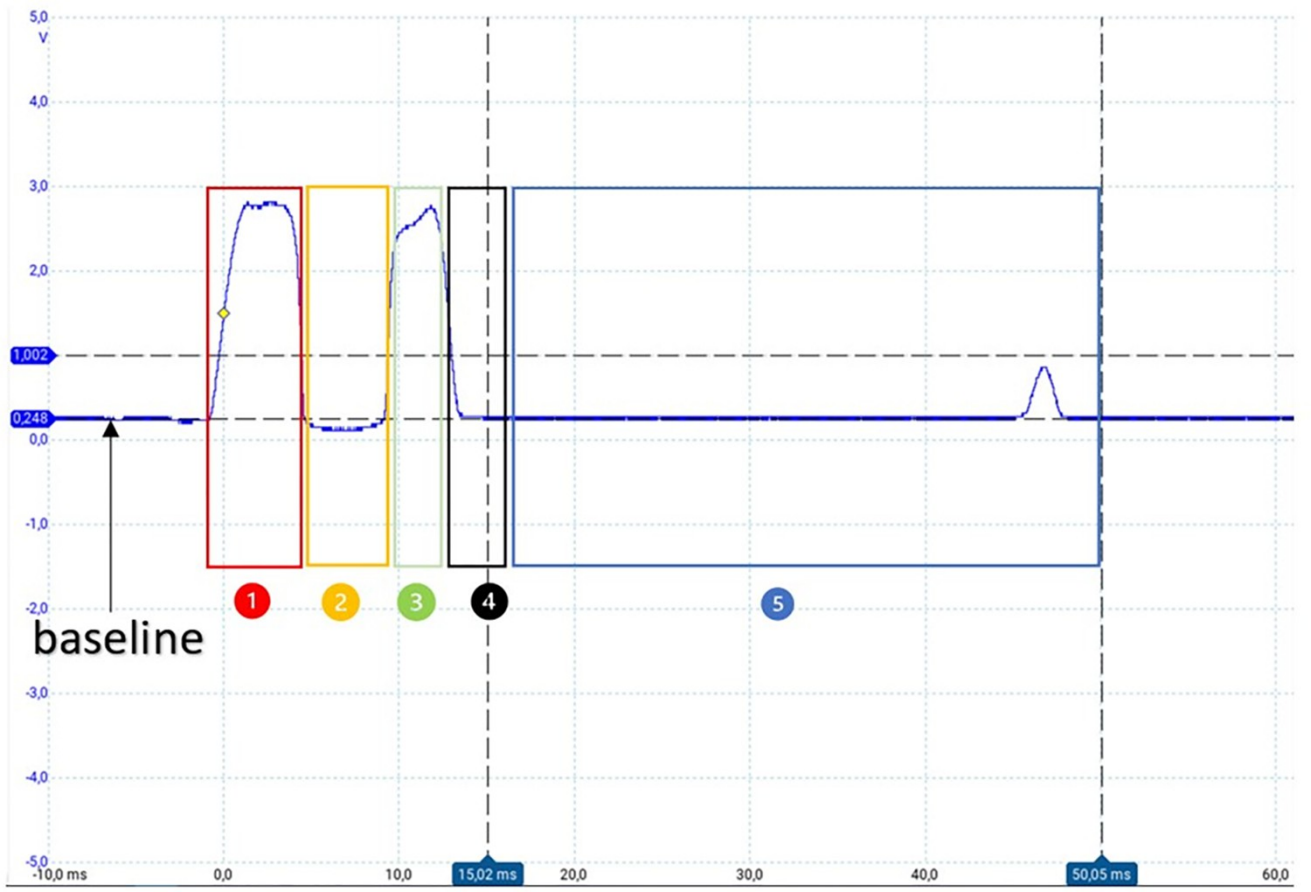
Pulse in detail of a normal drop. The numbers mark the different intervals.

Fig 5 provides a more detailed illustration of the pattern of a droplet that passes through the IR barrier. The voltage range typically varied between 0 V and 3.3 V, with an approximate voltage of 0.25 V obtained when the drop-counter was in place, which served as a baseline value for comparisons. This baseline was recalculated every time the urinometer was started but could also be manually calibrated from a menu.

To characterize a droplet, the voltage must first exceed an initial threshold of 1.5 V, followed by a waiting interval before a new voltage measurement is taken (interval 1). In interval 2, if the voltage is below a second threshold, which is the baseline in this case, we wait again for the voltage to surpass a third threshold of > 1.0 V (interval 3) and then wait for it to return to the previously established baseline value (interval 4). Once this is confirmed, it is determined that a droplet has passed through the infrared barrier. To avoid any bounce errors, a fifth waiting interval of 35 ms was immediately allowed to elapse so that the waveform could finish and restart.

In summary, our device has the capability to measure a single drop every 50 milliseconds, which equates to a maximum flow rate of 3600 ml/h, exceeding the requirements of our application. The thresholds and time intervals can be adjusted for improved performance through an ongoing pattern study and parameterization process. In this article, we present a simple pattern to aid in understanding the acquisition and processing concepts.

The S1 Fig depicts the flowchart for implementing the capture of drops that pass through the dropper. This flowchart can be redefined according to the needs to achieve improvements through programming.

**Fig 6:**
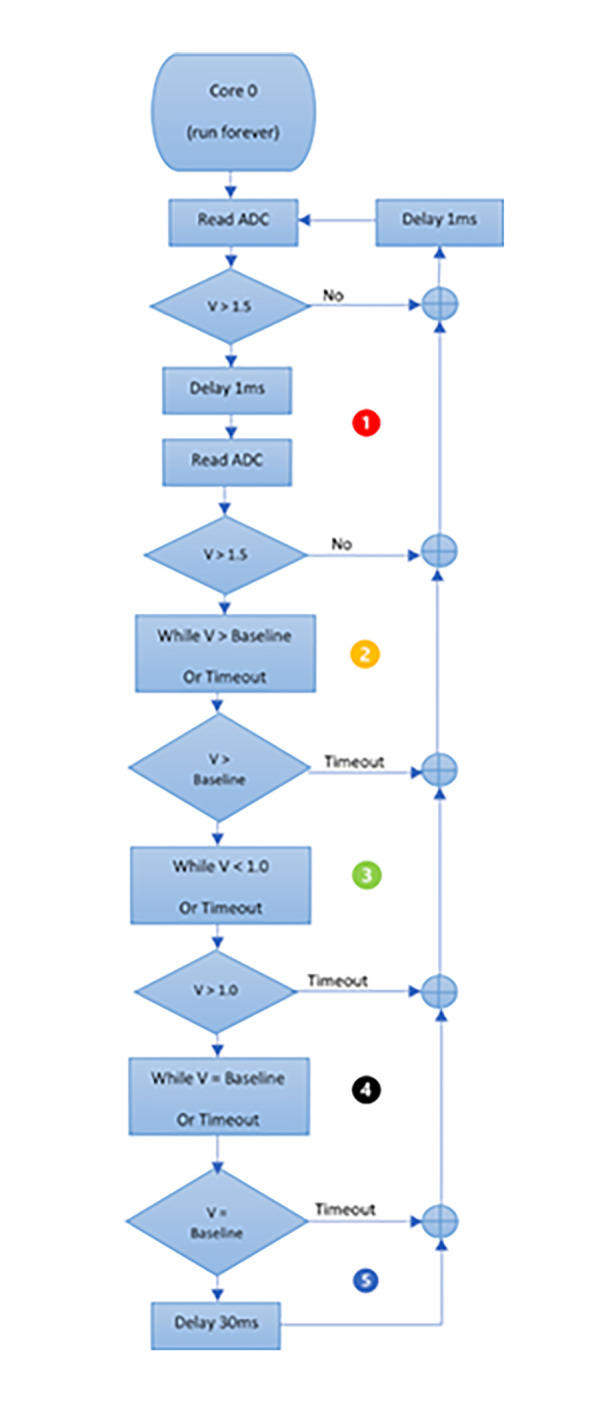
Waves of drops with varying degrees of inclination. (a) Narrowing observed in section 2. (b) As the inclination increased the value of section 2 increased and moved further away from the baseline. (c) Effect of approaching maximum inclination where the form disappeared.

Regarding the voltage in interval 2, it correlated to the vertical inclination of the dropper. In laboratory tests, we found that the device could tolerate a maximum angle of 5 degrees of dropper inclination. If the dropper was severely tilted, interval 2 disappeared, as shown in Fig 6. When the dropper was tilted less than 5 degrees, the wave time of the drop remained below 15 ms, which was typical for a normal drop. If needed, the narrowing of interval 2 could be measured, and an alarm could be added to alert of this situation.

**Fig 7:**
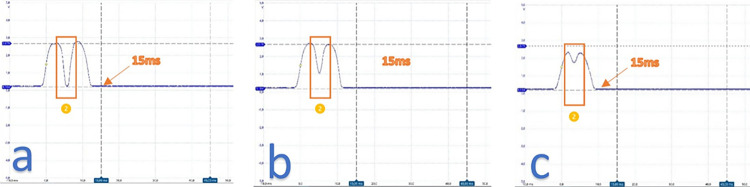
Jet-shaped droplet waves.

Fig 7 illustrates one of the detection patterns for continuous flow, also referred to as solid stream or jet. The jet extended beyond the baseline for a duration longer than 50 ms and concluded when the voltage returned to its baseline value.

When a jet was present, the waveform exhibited distinct differences. The initial interval was consistent, but from that point onward, the voltage values remained above normal levels for the remainder of the segment. Moreover, the jet persisted for a duration of at least 15 ms, and often exceeded the 50 ms window designed to detect drops.

From the jet pattern we could estimate the flow of urine. To determine the volume of urine, two factors were taken into account: the duration of the stream and the shape of the dripping tube. By analyzing these parameters, we could infer the amount of urine in the stream.

Our system was not designed to prevent the jet, but rather to detect this event through signal pattern recognition (Fig 7). When a jet was detected, it was possible to estimate the volume that had passed. This is different from other devices on the market that simply do not measure when the jet occurs. In S2 Appendix, we included details on the calculation of jet patterns. In the first version of the prototype, only the event was detected, leaving the estimation of the jet for future phases.

#### Central processing unit

The integration of an ESP32 microcontroller as a central processing unit (CPU) was aimed towards the application of IoT in general. This processor has two cores, allowing for one core to be dedicated to detecting light patterns gathered from the phototransistor, while the second core manages other firmware functionalities such as connectivity management (e.g. WiFi, BLE), information presentation, etc.

S2 Fig presents the system’s block diagram, showing that the sensor module feeds into the zero core. Additional functionalities are added around the CPU system.

**Fig 8:**
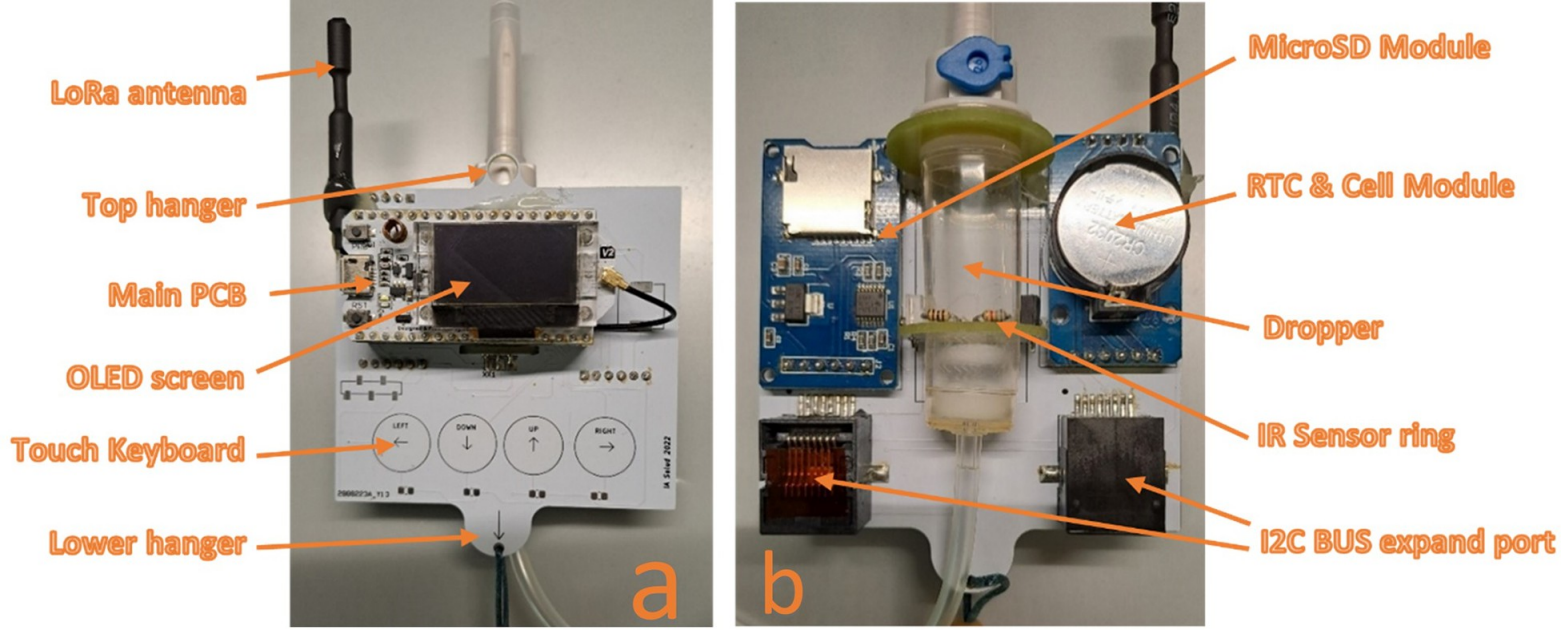
The prototype. (a) Frontal view. (b) Rear view with the location of the drop-counter.

Fig 8A and 8B show the front and rear views of the device, respectively. A development board (also known as the main PCB or printed circuit board) is present, containing the ESP32 (which already includes WiFi and BLE), the LoRa circuit, power management, and USB connection bridge for programming and cable communication. The board also features a RESET button, user button, and other systems for hardware operation.

#### Screen and keyboard

The device is equipped with an OLED screen and a keyboard to enable local visual representation of various data, measurements, alarms, and configuration menus. Examples of such menus and display screens are presented in S3 Fig.

#### Local data storage module

The system includes a local data storage module that utilizes a MicroSD card. While the capacity can vary, we recommend using an 8 GB card as we will only be saving data in CSV format. The module will maintain local log files of events, but under normal conditions, this information will be sent to the cloud. The local storage serves as a backup in case of communication failures, such as internet outages.

#### Date maintenance module

To ensure precise time management, we have added a real-time clock module (RTC) to the system. This feature allows the device to save date and time data to memory in the event of a power loss. The module comes with a CR2032 battery that can keep the date and time data accurate for approximately two months without power (see power supply below). As the device is expected to be on most of the time, this duration is more than sufficient.

Unlike other products that reset the flow data and record the flow over time without exact time stamps, our system records the exact day and time of the reset. Our goal is to provide continuous flow data with second-level precision and collect the data in a DD/MM/YYYY HH:MM:SS format.

#### Expansions module

Two RJ45 connectors have been included to enable hardware expansion through the standardized Inter-Integrated Circuit bus (IIC or I^2^C). This feature offers the potential to incorporate additional functions and avoid restricting the prototype to other testing options.

#### Printed circuit board

**Fig 9:**
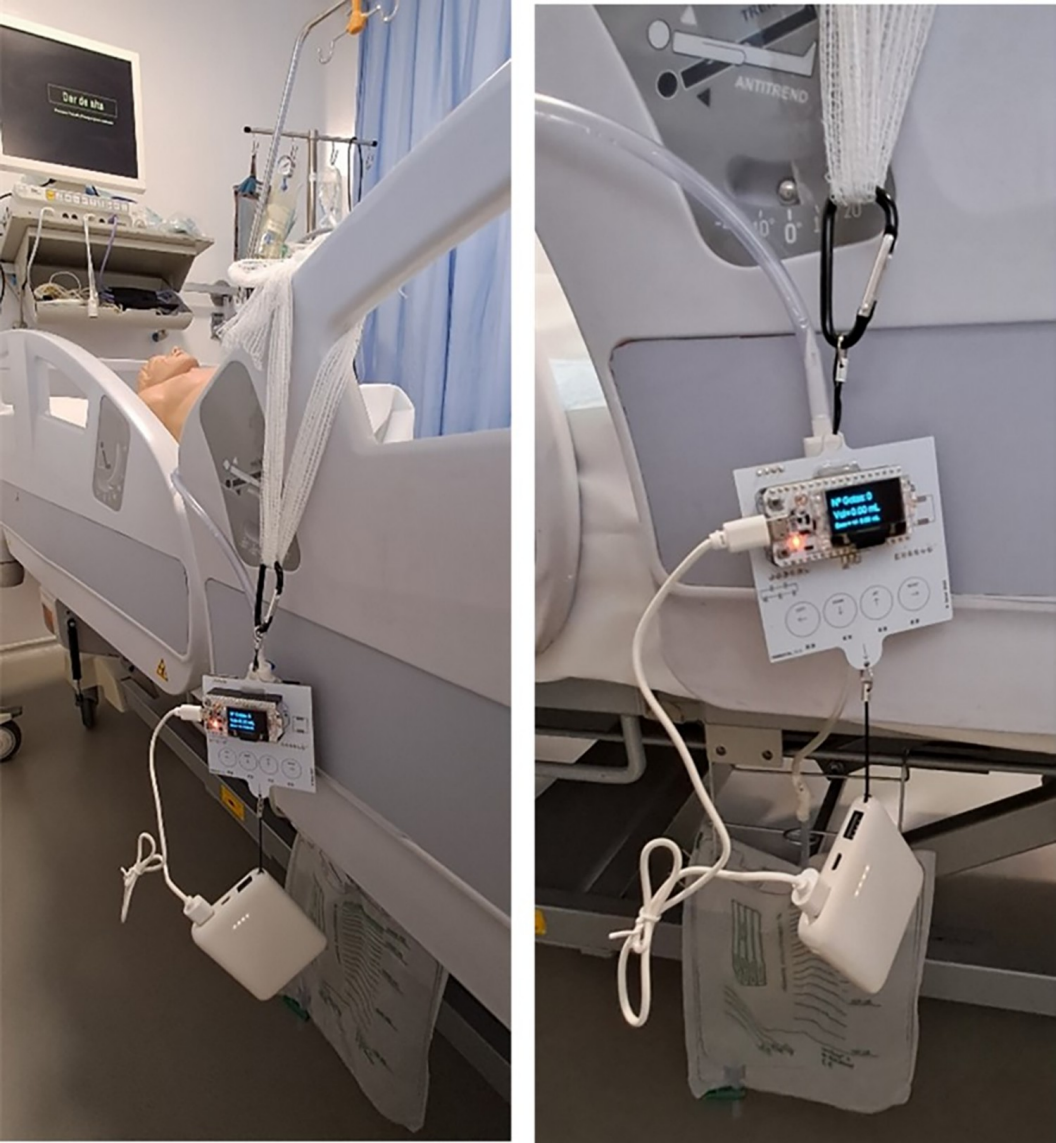
Two views of the device with the battery attached to the lower hanger.

The design of the printed circuit board (PCB) ensures that the assembly is mechanically oriented to remain perpendicular to the center of gravity. This is necessary to maintain the necessary verticality for the dripper to function properly. The plate was designed to be hung preferably from the patient’s bed. The lower hanger was used to attach the rechargeable battery, which kept the dripper in a vertical position by using its weight. Fig 9 displays the device with the battery attached to the lower hanger, which forces the device to remain upright.

#### The power supply

The device has a 5V power supply with a consumption not exceeding 100mA (maximum measurement in tests of 50mA, without transmissions). For mobile use and to eliminate dependence on power cords, the power is provided via batteries or rechargeable cells. The choice was made to utilize rechargeable batteries due to their ease of use and lack of waste production. Within the range of rechargeable batteries, a power bank was selected. Although it met all the requirements of our device, looking towards future commercialization, it would be necessary to identify a medically certified power bank (not currently commercially available) or substitute it with a certified battery.

The battery calculations were conducted while the device was always on and the OLED screen was not turned off. It has been determined that a 5,000 mAh battery can power the device for a maximum of 100 hours or 4 days, while a 10,000 mAh battery can last up to 8 days. After this duration, the battery needs to be charged or replaced. It is recommended to recharge or replace the battery when the patient replaces the Foley probe, which typically occurs every 3–7 days.

On the other hand, for battery recharging, we propose to replace it and perform the charging cycles outside of the ICU environment to minimize risks associated with battery safety.

#### Mechanical considerations for the prototype

The electronic unit has an embedded casing design that enables its functionality while also saving on costs by eliminating the need for an enclosure. To prevent any contact with humidity and to meet the galvanic isolation standards, the circuit is electrically isolated using specialized coatings. This aspect of the device is particularly advantageous in terms of cost savings. The device has small dimensions of 8 cm x 7 cm x 4 cm and weighs approximately 200 g without the battery.

#### Wireless communications

The inclusion of LoRa communication technology enables efficient communication optimization in the broader IoT environment. Specifically, when applied to Internet of Medical Things (IoMT), it results in an energy-efficient, low-cost, and effective wireless communication strategy over long distances. As a result, the device is open to various types of connectivity, making it easy to select the most suitable option for a specific situation or moment.

#### LoRa

Up until now, wireless devices, such as the one proposed, have relied on Wi-Fi/BLE connections which have limited communication ranges. Our proposal is distinct in that it utilizes LoRa communication, specifically the LoRaWan protocol, which eliminates the dependence on gateway distance. This simplifies the network infrastructure and reduces costs, as fewer access points are necessary. It is important to note that in hospital facilities, distances can be significant, often exceeding the maximum range achievable with Wi-Fi/BLE technology (usually around 100 meters). Additionally, the hospital environment is unique in that it has areas covered in metal for insulation, which can impede the propagation of wireless signals. As a result, repeaters are commonly used, often at close proximity. Implementing a LoRa system would provide a more economically viable infrastructure by eliminating the need for numerous repeaters. It would also prevent network saturation caused by a multitude of devices, which could hinder the effectiveness of the Wi-Fi network used for other high-volume data transmission tasks.

Furthermore, in hospital settings, communication range can often be severely limited by walls and other barriers, but several studies have shown that LoRa transmissions outperform other methods in such environments. LoRa technology can greatly increase communication distances, allowing for transmission over several hundred meters or even kilometers, depending on indoor conditions. Additionally, LoRa enables low energy management, and data security is ensured through compliance with protocol standards. The use of LoRa technology requires hardware that is not overloaded in the devices, and data is further protected by being pseudonymized, limiting access to only researchers and developers who understand the data. Multiple 64-bit and 32-bit protection keys are also in place for added security.

The device can be used locally or remotely and can send alarms or data for connection to the IoMT platform. Its purpose is to serve as an IoMT device to support clinical decision-making. Implementation of the LoRaWAN network can also reduce infrastructure costs, as the urinometer only needs to send small amounts of data at specific intervals rather than continuously.

LoRa communications and the LoRaWAN protocol have multiple layers of security, ensuring that only authorized data is collected, and that encrypted data is transmitted to the cloud or within a private, local network for added security. In this study, the nodes (urinometers) were connected to a gateway, which served as a bridge between the LoRa network and another protocol, usually Wi-Fi, to transmit data to a cloud platform (as shown in S4 Fig). The device was programmed to send urine volume data every minute, and the dashboard displayed the information according to clinical requirements (e.g. every 10 minutes).

In our initial LoRa communication trials, Class A was employed as the goal was to achieve the lowest possible energy consumption, with the urinometer initiating or conducting the communication. Although this class allows for a waiting period for reception, this feature was not required in the initial stages. The device does not need to send data in real-time, even though we refer to it as real-time diuresis, the data is sent minute by minute or even every 5–10 minutes (depending on the clinical context). The device also initiates communication when an alarm is activated.

An example is provided of a sent data frame, which typically occupies only a few bytes (never exceeding 255 bytes) according to the specifications of the LoraWan protocol.


FRAME=28/2/2023;7:6:32;15;0.75;0.08;0.24;1;0.00 (2)

S5 Fig displays the dashboard, which collects data from the urinometer.

#### Pseudonymization method

Up to this point, we have focused on the design and development of the device. We have not yet reached its use in actual patients; therefore, although we have recognized the need for pseudonymization, we have not developed it yet. However, in the prototype, we have identified the patient by bed code, not by personal identifiers. This model could be easily modified according to the user’s needs. If, for instance, a specific ICU has a centralized and private data lake (local server in ICU), pseudonymization would not be necessary. We believe, therefore, that these usage details will depend on the end-user and their IT infrastructure.

In addition to pseudonymization, it is crucial for the device to implement additional security measures, such as data encryption in transit and at rest, secure access to data, and the adoption of robust data management practices such as data minimization and anonymization, to ensure comprehensive protection of privacy and personal data. All of these are fulfilled by the UrinAI, thanks to the secure LoRaWAN protocol.

#### Colorimetry

Health care workers commonly observe visually the color of a patient’s urine to identify potential conditions such as hematuria, which is indicated by a red color due to blood in the urine and can suggest an infection, or jaundice, which causes a yellow color due to bilirubin accumulation in the urine. However, this method is subjective and can vary between individuals, especially if they have color vision deficiencies. To provide a more objective measurement, a sensor has been developed to automate this process. Initially focused on detecting hematuria, the sensor can detect different shades of color. It should be noted that if urine is not properly preserved within 2 hours by refrigeration or chemical preservation, its properties including color can be altered and therefore may not provide a reliable basis for analysis [48]. This can happen when observing urine stored in a urinary bag throughout the day.

Our urinometer presents a solution to the aforementioned issue. The TCS34725 model sensor we employ is capable of providing various color measurements, but we utilize the RGB format measurement along with color temperature data and LUX received by the sensor, as well as unfiltered light reception (i.e., all channels together without a filter). An example is provided of a sent color frame.


COLOR=R:177/G:255/B:254/Clear:65535/C_TEMP:11325/LUX:40712# (3)

Moreover, color analysis reinforces the device’s safety. In the event that we detect color in the dropper chamber (liquid in the drain probe) and the flow sensor does not yield a result, we can trigger an alarm indicating a potential issue, such as a system obstruction.

**Fig 10:**
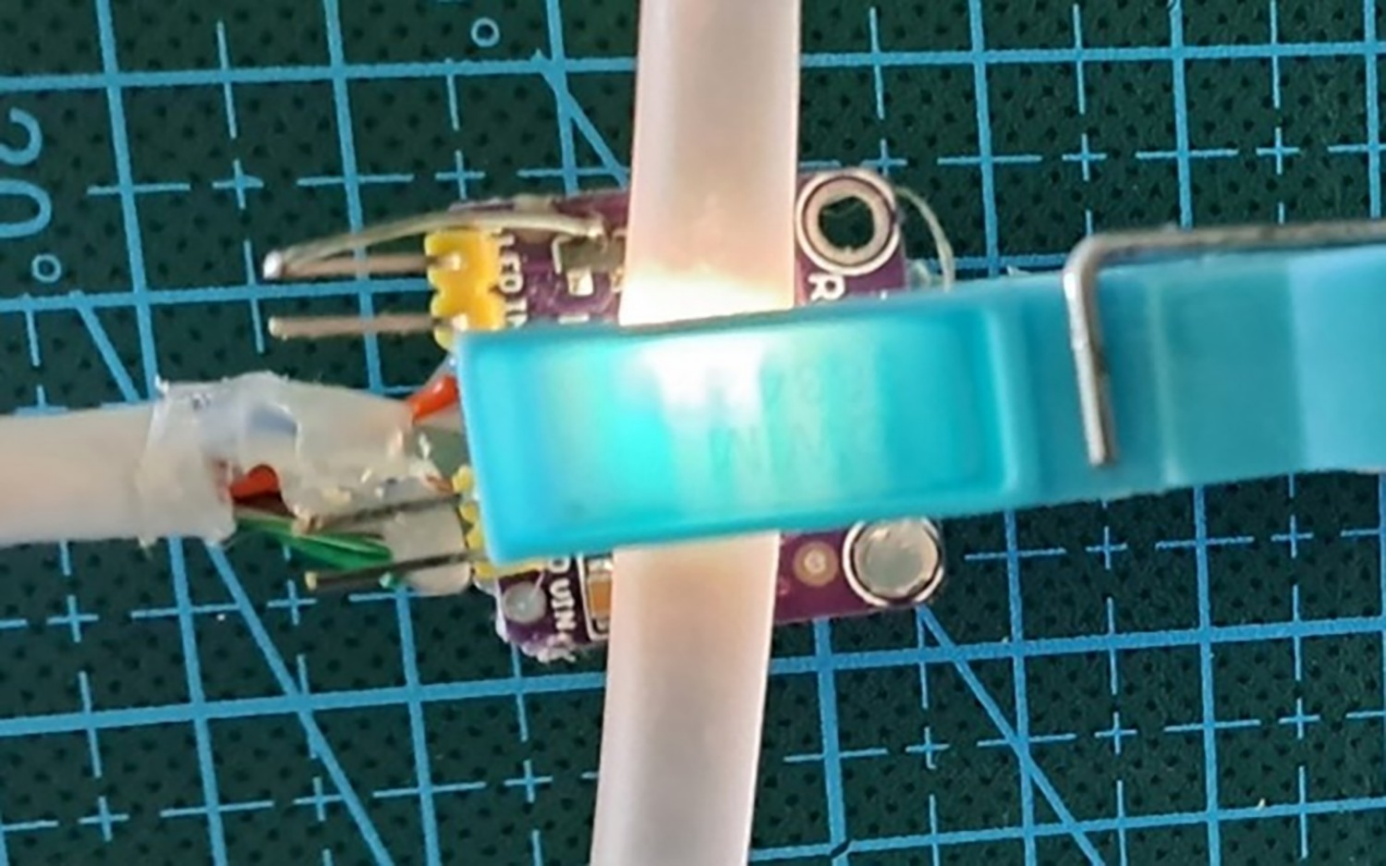
Prototype of the colored probe in clip format to be attached to the drainage tube prior to passing through the dripper.

The colorimetric sensor is also removable and is automatically detected when connected to the I^2^C BUS expansion ports. This clamp type sensor is connected to the drainpipe (before passing through the dripper) and detects color differences, as shown in Fig 10.

The hue of urine can range from transparent to nearly opaque black. Scientific research indicates that urine color can be a useful indicator for identifying various clinical pathologies [48]. Furthermore, the inclusion of urine color in urinary flow measurements can provide valuable insights for physicians.

#### Alarms

In this prototype phase, which emphasizes measurement, we only specify a few types of basic generic alarms. The urinometer is designed as a programmable device, customizable to the needs of each patient and clinician. As the prototype progresses and clinical tests are conducted, these alarms will be better defined.

For the time being, the following basic default alarms have been proposed:

1. Alarm for when the flow decreases below 0.5 ml/kg/h, signaling a "risk of renal function deterioration."
2. Alarm for when urine measurement abruptly ceases, indicating a "risk of obstruction."
3. Alarm when the bag is nearly full ("bag fill alarm > 1500ml").
4. Colorimetric alarm detecting red as a "warning of possible hematuria.

#### Costs of the prototype

Two prototypes have been made at a material cost of €60. Our target cost for mass production is €15 to €20. The outcome is an economically viable device in terms of material costs, combined with the use of commonly available disposables in hospital environments. A detailed bill of materials can be accessed in S1 Table.

#### Preparation of a realistic simulation model

The prototype’s operation is verified on real time using two techniques. An ICU manual urinometer (Ureofix® 500 model) is used to compare our urinometer measurements. The first technique involves functional tests from a physiological point of view. To do this, a model of the urinary system was constructed, consisting of a jar that simulated the kidney, a flow regulator to preset a flow rate for the ureters, connected to a nitrile glove as a bladder. A Foley catheter was inserted into the bladder, which connected to the drip chamber of our urinometer. The outlet of the urinometer connected to the urine bag. This simulator reflected the conditions of gravity and was highly realistic.

The second technique allowed us to verify the flow ranges and pressure conditions that may occur. For this technique, tests were carried out using a precision peristaltic pump (Braun Infusomat Space), in which we preset a specific flow and volume and compared the measurements displayed by the urinometer.

S3 Appendix includes details of the recreation of the testing system and the pump model.

## Results

### Laboratory tests

The tests conducted using the urinary system simulator demonstrated that the system functions physiologically and is capable of detecting the passage of droplets. To test different flow ranges and densities, a perfusion pump was used. Urine has a density ranging from 1 g/ml if the patient has consumed a lot of fluids, up to 1.034 g/ml if the patient has consumed almost no fluids. A normal range of urinary density is between 1.005–1.030 g/ml.

The Braun Infusomat Space is a widely used medical device for the administration of intravenous fluids and medications. The Infusomat Space typically has an adjustable flow range that can vary from 0.1 ml/h to 1200 ml/h. The flow accuracy is usually in the range of +/- 5% or better.

To validate the accuracy and reliability of the UrinAI measurement capabilities, the following methodology was implemented:

1. Experimental Setup:
  - The Infusomat Space line was fed with the test liquid, having a specific density within the range of densities studied.
  - The output fed into the drip system, where the UrinAI measures.
  - The drip system, in turn, fed into the urinary bag.
2. Methodology:
  - A target volume and a specific time for the infusion were set.
  - The urometer was zeroed.
  - The infusion was initiated in the Infusomat Space.
  - Upon reaching the target infusion time, the volume measured in the UrinAI was recorded.
3. Result Analysis:
  - The volume set in the Infusomat Space was compared with the volume recorded in the urometer.
  - The difference between the two volumes was calculated.
  - The experiment was repeated several times to obtain an average of the differences.

To test the behavior with different liquid densities, a volume of 100 ml was set on the infusion pump and the density was varied. The different densities tested correspond to the use of 0.9% physiological normal saline with a density of 1.005 ml/g, seawater with a density of 1.025 ml/g, and normal water with a density varying between the two aforementioned liquids.

**Fig 11:**
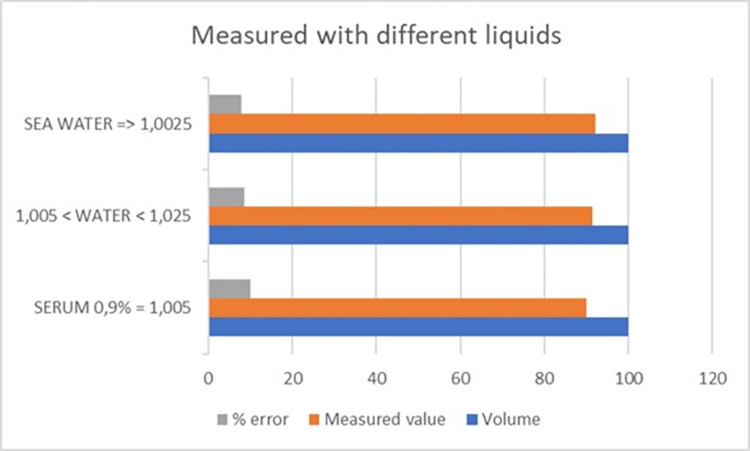
Value measured versus standard volume with varying densities.

These tests showed that the dropper was capable of assimilating different densities. The results indicated that the measurements of the device vary slightly with the varying density, but this was not significant because the error remained within similar margins, as shown in Fig 11.

Different flows and volumes have been tested with the infusion pump to verify accuracy, measurement repeatability, and deviations. Over 100 tests have been performed, changing flow and volume ratios.

**Fig 12:**
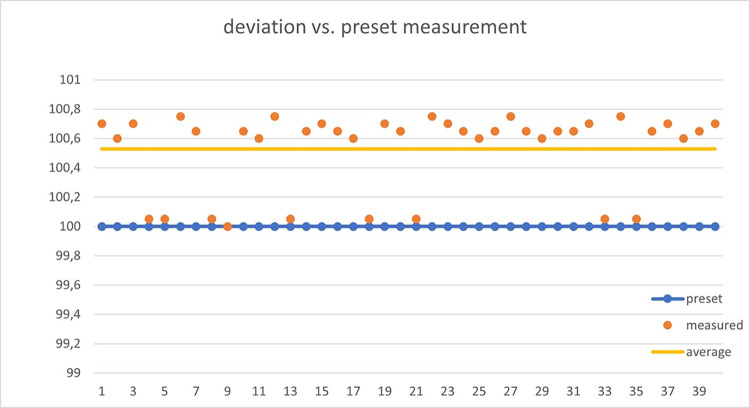
Deviation of volume measurements when repeatedly applying 100ml volumes using the infusion pump.

Our device relies on the inherent error in the dropper, which is typically around 10%. The dropper is specified to measure 20 drops as equivalent to 1 ml, with an accuracy of ±0.1 ml. Figs 11 and 12 demonstrate how the obtained results do not exceed the dropper’s margin of error when assessing the measured volume.

The tested flows range from low flows of 1 ml/h up to maximum flows of 500 ml/h. Clinically, it is possible to exceed this flow rate if the patient has potomania (an impulsive desire to drink large amounts of water), but if this were the case, the clinician would be obliged to use maneuvers to reduce diuresis. For critical patients and initial tests, a flow limit of 500 ml/h was considered acceptable. The device performed exceptionally well at low flows, which is a major problem for urine absorption in critically ill patients, while there was more deviation at higher flows (above 200 ml/h). In critically ill patients, it is essential to control low flow and act with sufficient time in case of possible renal failure.

A systematic error of approximately 10% was detected (due to dropper error). This error increased as the flow rate increased. Since the error was systematic, it could be automatically corrected, resulting in a final overestimation error of no more than 15%. Therefore, the device had the required accuracy for our purpose of measuring trends and detecting low levels of diuresis. It should be noted that, according to our clinicians, trend information on diuresis is more useful than a specific value. The device is designed to provide critical alerts when the flow is excessive (above 300 ml/h) or very low (below 30 ml/h). In S2 Table, the measured values are summarized in comparison to those pre-set in the infusion pump. Fig 12 shows the deviation in different tests where the pump was preset to 100 ml.

**Fig 13:**
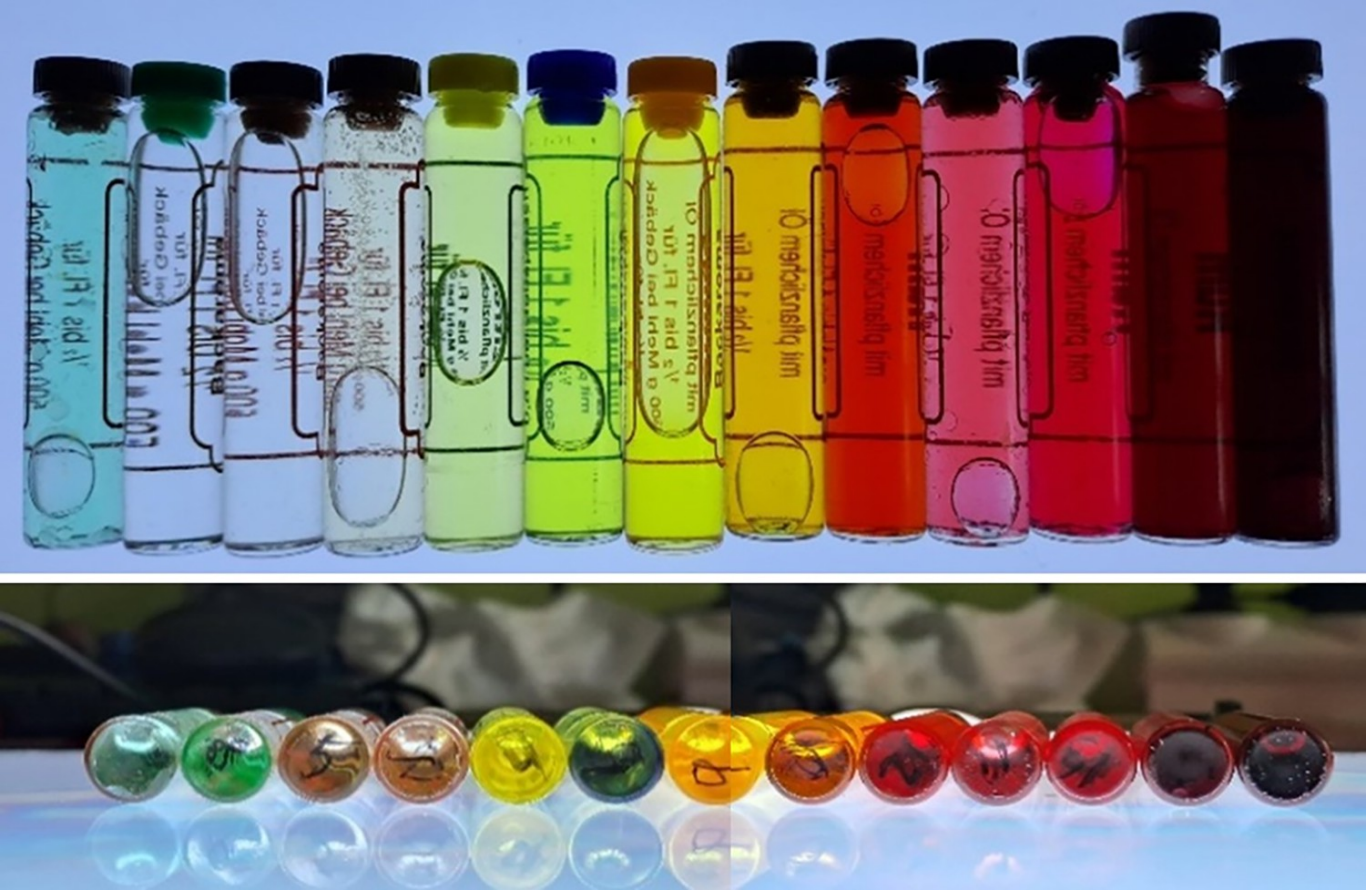
Color samples to perform the first color tests.

In laboratory colorimetry tests, samples of different colors were used (Fig 13) and it was verified that the device detected the presence of the tube, the liquid inside the tube, and the color of the liquid.

The TCS34725 color sensor is a digital RGB (red, green, blue) color sensor that provides an accurate color measurement within 4.4%. It can detect light wavelengths in the range of approximately 400 nm to 700 nm. This range encompasses the visible region of the electromagnetic spectrum. It has a resolution of 16 bits for each color channel (red, green, blue, and clear). The sensor is designed to function under low light conditions, enabling accurate measurements even in environments with dim lighting. It incorporates an IR filter to prevent infrared light interference, thereby enhancing the accuracy of color measurements.

To test the capability of the TCS34725 sensor to detect different colors and hues, the following methodology was used:

1. Environment Preparation: We created liquid samples to obtain a variety of colors (see Fig 13). These samples were prepared by mixing water with different dyes. Colors associated with various types of urine (straw-yellow, greenish, orange, yellow, red, etc.) were created. These samples were packaged in a glass tube to simulate the fluid inside the tube feeding the dripper, where the color was actually measured. The sensor was arranged in a clamp that holds the tube perfectly, and there was a neutral white light, ensuring that the ambient light is suitable and constant during the measurements.
2. Sampling and Measurement: We placed the sample in the sensor and recorded the obtained data. The colorimetry data included information on RGB values and potentially other parameters such as color temperature. We then applied these values in an RGB color viewer (we used the free COLORMANIA software, but any software displaying color would work).
3. Data Analysis: We compared the colorimetry data obtained from the different samples and observed variations in RGB values and other parameters to identify different colors and hues. We performed a visual test by comparing the sample color with the one shown by the viewer when entering the RGB values provided by the sensor.

**Fig 14:**
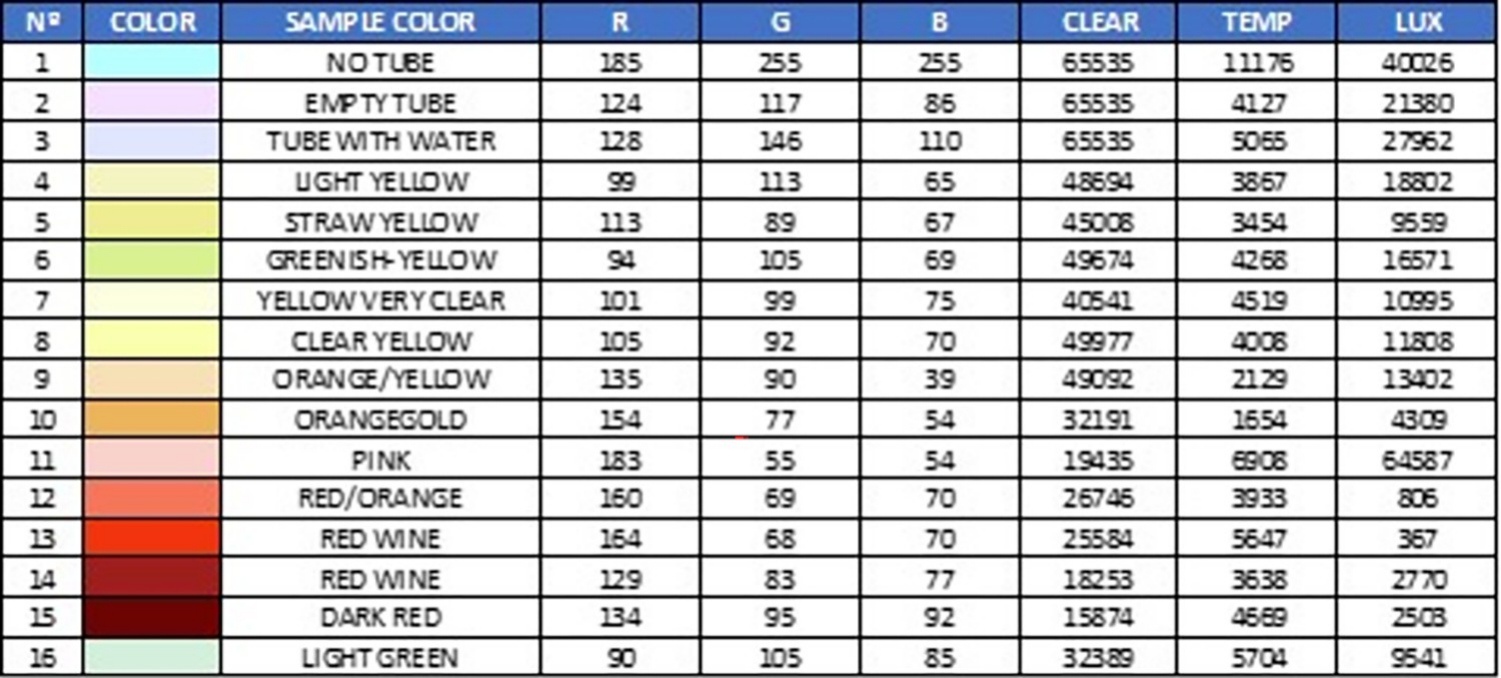
Different measurements with the values obtained by the sensor.

The Fig 14 demonstrates that the sensor is capable of discerning between different colors, opacities, tonalities, and brightness levels.

Additionally, we verified that the sensor was sensitive to small changes. When the sensor was not placed, it registered a cyan color that corresponded to the clamp’s background color used in the prototype, as shown in Fig 10. S6 Fig show the dashboard screen for color test.

The typical error in measuring the RGB values of the TCS34725 sensor falls within the range of +/- 2% to +/- 5%. This implies that the obtained measurements may deviate by as much as 2% to 5% compared to the actual color values. Some tests carried out in other studies [49] concluded that the sensor differentiates a change of 10 points in 8-bit RGB values, which amounts to a resolution of 4.4%, with a repeatability of 2.2%.

In our preliminary tests, we only aimed to distinguish very specific colors, and the table we present helps to illustrate our ability to differentiate primary colors and observe measurement differences in certain color hues. For initial alarms, such as detecting hematuria, this is sufficient. In subsequent phases, more alarms or measurements can be implemented.

Finally, after activating LoRa connectivity, we verified that the data arrived correctly at the local server, as expected. In the future, it will be possible to process the data on the platform to analyze the information as deemed appropriate by the clinician (e.g. alarms, pattern detection, and predictive models).

### Operational summary

**Fig 15:**
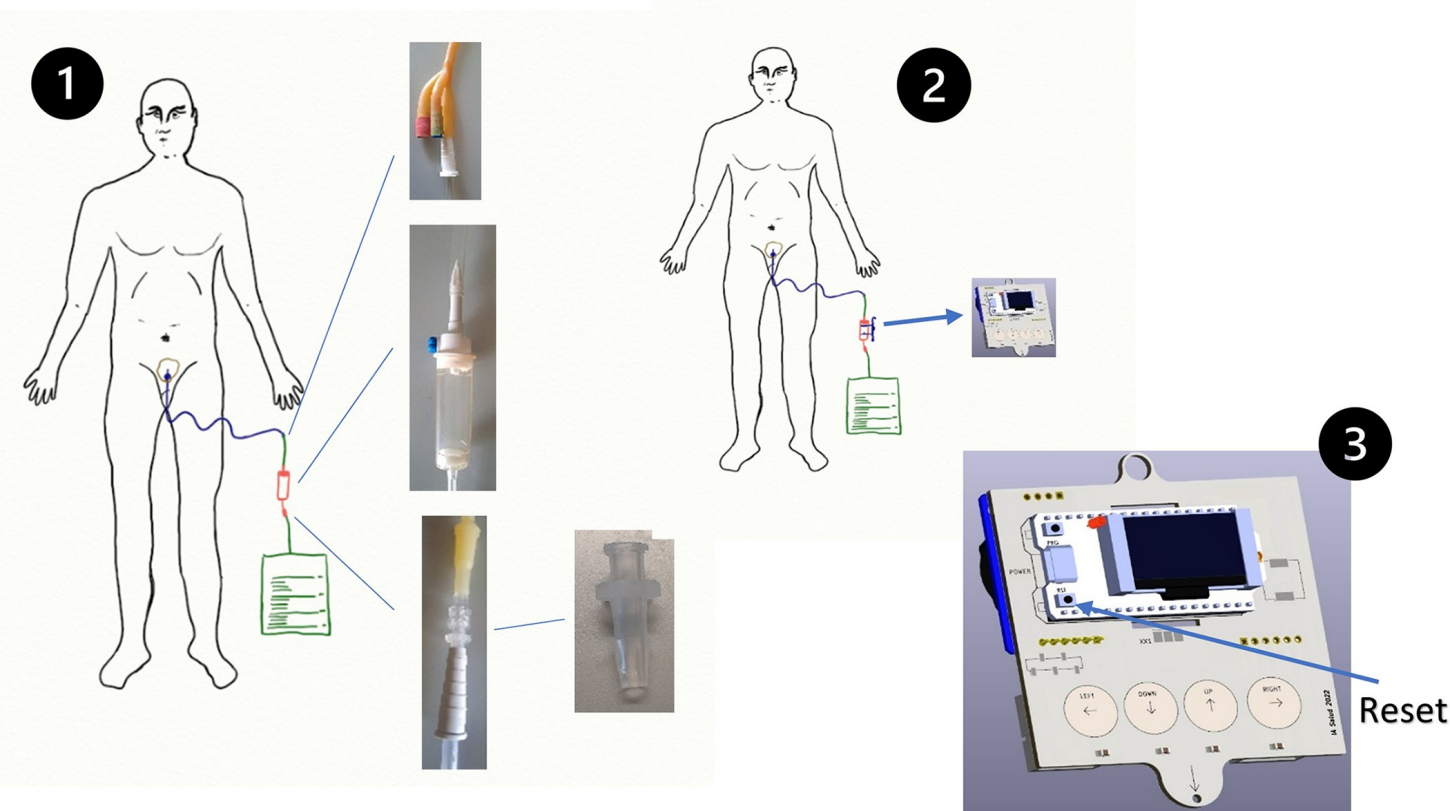
Getting started procedure.

The basic operation of the UrinAI meter is simple. First, we need to place the urinary catheter on the patient according to the protocol provided in the hospital center. The bladder catheter will be connected to the drip and the outlet of this with the urine bag. In Fig 15, we can observe the different adapters of the system. The second step is to place the urine meter around the drip. Once the urine meter is turned on, if necessary, patient parameters such as weight and bed number, alarms, etc., can be configured. When everything is ready, by pressing the RESET button, the urine meter will start recording a start date and counting the drops that pass through the drip. The screen presents the information locally, and every 10 minutes, the urine meter sends a package of information to the local server through LoraWan. This information is presented on the panel that can be accessed from different devices. In case of a pre-set alarm, such as a bag about to be filled or detection of hematuria, etc., an alarm message is sent, and there may be the possibility of having a local acoustic alarm.

The urinometer displays real-time information on the screen every second. All events are recorded on a MicroSD card in CSV format. This means that in case of communication failure, we have a record of the measurements and alarms. The operation of the urinometer can be seen in several videos available in the supplementary material.

Every 10 minutes, the urinometer sends diuresis information to the server. We understand this interval to have clinical significance, although this value is adjustable. In addition, a color sample is taken at the same interval.

If liquid is detected in the upper tube but no drops fall, an alarm is activated (device blockage warning). An alarm is also activated if a specific pre-defined color, such as red, is detected to indicate possible hematuria (blood in the urine).

In the event of a serious event such as anuria (total renal function failure), an alarm is instantly sent to the server to alert the clinician through the dashboard.

Finally, if the device fails (fails to record or send values, has no battery, is too tilted, etc.), the clinician will be alerted that it is not functioning, but can still manually perform measurements (volume and color) because we still have the traditional urinary bag measurement system available.

S1–S3 Videos show examples of how the urinometer works and how to access some functions from the menus.

## Discussion

Our device is designed to meet the requirements gathered from the clinical needs of the intensivist physicians we collaborate with, and to overcome cost barriers. In summary, UrinAI distinguishes itself through the simplicity of its design, utilizing commonly available materials, and is specifically targeted towards an Internet of Medical Things (IoMT) environment with the concept of ease of use for clinical staff. Additionally, it incorporates colorimetric measurements. Unlike market devices, UrinAI does not require additional disposable elements and can be used with components already available in hospitals such as droppers and urine bags. The latter, being standardized, can be used from any brand or manufacturer.

We have described the design and construction of an automatic urinometer connected to the cloud that is simple to use and economically viable for mass production. The device is designed as a decision-support system, helping clinical staff save time.

Compared to available commercial products and proposals in scientific literature, our device offers eight distinctive features. Firstly, its low cost (€60 compared to €2,000 for available commercial products), not accounting for the potential cost reduction in mass production. Secondly, the use of low-cost disposables that are widely available (compared to high-cost proprietary disposables). Thirdly, its small size and ease of use (compared to bulky and space-consuming devices in an ICU). Fourthly, the incorporation of long-range and low-power wireless connectivity (LoRa) (compared to WiFi and BLE). Fifthly, the incorporation of a clock for processing time-variable data points (compared to measurements relative to the start time of use). Sixthly, the incorporation of a colorimeter for urine color analysis. Seventhly, in case of device failure, the manual measurement on the urine bag is still possible due to its design. Lastly, as there is no direct contact with urine, the probability of retrograde contamination associated with our device is nil.

One of the factors complicating the use of automatic urinometers, in addition to the price, is the use of proprietary disposables. That is, each device necessitates the use of a specific urinary bag or other specific elements (in some cases, even the catheter). This in itself is not a problem. However, not utilizing these elements carries many advantages, in terms of scalability, ease of management, and cost.

The requirement for specific materials for the urinometer compels double orders and complicates the purchasing logistics for the medical center. If an incident occurs, it is not possible to use components from another manufacturer or distributor.

In fact, reviews conducted on several discontinued market models precisely point to the failure of the project due to the cost of both the device and the disposables.

Therefore, our urinometer is proposed without the need to use any type of proprietary disposable. By using a standard intravenous infusion dropper as the element to be added, and a standard urinary bag, we avoid this issue. The standard dropper commonly used for intravenous infusion measures 20 drops per 1 ml, differing from micro droppers (60 drops = 1 ml) or other types of droppers such as those used for blood infusion.

Our urinometer does not need to be sterilized or disposed of after use, as it does not come into contact with the patient’s fluid or the patient themselves. It is an external element without direct contact. Nevertheless, like any electronic device, if we wish to clean it, it can be sterilized using a UV light chamber (a common practice) or even ultrasound cleaning, which is a routine cleaning system in electronic devices.

For now, our urinometer is far from the commercialization phase, so these aspects are being considered for future stages. The use of certified protocols and the potential for adaptation through programming make its implementation viable in future phases. In its current stages, the urinometer uses secure protocols (LORAWAN) and does not use real patient information in any case.

It is important to consider that regulations and requirements can vary depending on the country and the healthcare system. Therefore, it is essential that the offered device can adapt to the requirements and needs determined by local experts (e.g., GDPR in the European Union or HIPAA in the United States, or PCI DSS or ISO27001), as well as interoperability requirements (e.g., HL7 format or FHIR standard), integration with electronic medical records (EMR), and secure data transmission (e.g., TLS, VPN). Our device can adapt to all of these without any problem and according to needs.

On the other hand, we should consider the following limitations. Although normal urinary diuresis ranges between 1005 and 1030 g/L, and we have conducted tests with saline (1005 g/L), seawater (1027–1030 g/L), and drinking water (1005–1030 g/L), it would be necessary to extend the tests to more extreme densities and to different points of previously calibrated intermediate densities.

The proposed device has been tested in laboratory conditions simulating patient scenarios. It would be necessary to conduct a validation in ICU critical patients in an initial phase, and subsequently in hospitalized and home-care patients to verify its proper functioning under such conditions.

Another limitation is the lack of certification for UrinAI as a medical device. However, we are still in the early phases of its development, and such certification will be the subject of future work, once we have demonstrated its functionality and feasibility under laboratory conditions.

In the future, we will work on validating the colorimetric function with standardized colors, not only with visual classification. Moreover, the validation of the colorimetric function with real patient urine remains pending, specifically with pathological urine discoloration such as hematuria (blood in urine), jaundice (bilirubin in urine), etc.

Improvement in the form of a clamp for the drip holder to be used with different standard drips that may vary slightly in diameter, and improving the color sensor’s placement, or investigating ways to further integrate it, are design aspects with potential for enhancement.

Another pending task is improving the capture and measurement of the fluid as it passes in jet form.

We should bear in mind that in UrinAI we must maintain it vertically, with inclinations not exceeding 5° (achieved successfully thanks to the weight of the battery), while keeping the system below the patient and above the urinary bag (gravity system). It could malfunction if these conditions are not met.

One of the weak points of the device is its measurement error of around 5–10% with maximums of 15%. However, from the clinical perspective, detecting changes in trend and cessation of urine production (anuria) is more important than exact urine output measurement. Although there are commercially available products that measure urine output more accurately, they are not viable for use in our hospitals’ ICUs.

Lastly, our device does not work well when the urine flow is high (>500 ml/h). The main cause of this is the filter in the dropper that impedes the normal flow of such large amounts of urine. A possible solution in the future is to use droppers without filters. However, such high urine output in ICU patients is rare, and the likelihood of renal failure due to anuria is very low (as patients are in polyuria). Clinicians should be informed of this limitation when offering them the device.

The implementation of UrinAI in the ICU can offer the following benefits:

1. Time and resource savings: The automation of the process of measuring and recording diuresis and colorimetry through the UrinAI can save time and resources for the nursing staff (about 8 hours a day for a nurse and for a 10-bed ICU: 15 beds * 24 measurements a day * 2 minutes per measurement = 12 hours).
2. Accurate and reliable measurement: They eliminate the need for manual measurements, which can reduce errors and increase consistency in data collection and recording.
3. Real-time diuresis monitoring: This provides an accurate and up-to-date view of kidney function and patient fluid balance with early detection of renal function deterioration and ease and accuracy in measuring daily fluid balances. Nursing staff and doctors can easily access this data and properly track the patient’s response to therapy and changes in their clinical status. They can therefore make decisions based on objective and real-time data. This can help optimize patient care, adjust fluid therapies and medications more accurately, and assess the patient’s response to treatments such as the assessment of renal reperfusion in shock patients.
4. Colorimetry for early detection of problems: Changes in the color of the urine can be indicative of certain problems related to the urinary system, such as infections, bleeding or metabolic imbalances. Real-time monitoring of colorimetry can help detect these alterations early and allow for swift and appropriate intervention.
5. Quick detection alarms for all of the above and urinary catheter obstruction.

The implementation of an automatic urinometer in intensive medical care can provide continuous and accurate monitoring of diuresis and colorimetry. This can improve patient care, facilitate decision-making based on objective data, and potentially enable early detection of problems related to the urinary system. These benefits can contribute to more effective care and better management of patients in intensive care.

## Conclusions

In summary, due to its low cost, time-saving automation of data, simplicity of infrastructure, and use of common disposables, this device provides a path for incorporating IoMT in urine output measurement for catheterized patients and allowing for widespread use in hospital clinical settings. The next step is to validate its use in real patients in a hospital ICU environment (we already have ethical approval to start the validation study with real patients).

## Supporting information

S1 Fig Flowchart outlining the process by which the software determines the count of droplets. (TIF)

S2 Fig General block diagram of the System to be implemented. (TIF)

S3 Fig Various menus and information on the screen. (TIF)

S4 Fig LoRaWan diagram block. (TIF)

S5 Fig Control panel hosted on a local server that receives data from the urinometer. (TIF)

S6 Fig Screen in the color sensor dashboard. (TIF)

S1 Table Bill of Materials. (PDF)

S2 Table Data record. (PDF)

S1 Appendix Physiological and clinical data. (PDF)

S2 Appendix Jet detection. (PDF)

S3 Appendix Realistic simulation model. (PDF)

S1 Video Urinometer. (MOV)

S2 Video Patient menu in urinometer. (MOV)

S3 Video Menus examples for urinometer. (MOV)
