# Supplementary figures and images for: Development of a urinometer for automatic measurement of urine flow in catheterized patients
Source: PLoS One. 2023 Aug 31;18(8):e0290319. doi: 10.1371/journal.pone.0290319 (PMC10470914; doi:10.1371/journal.pone.0290319)

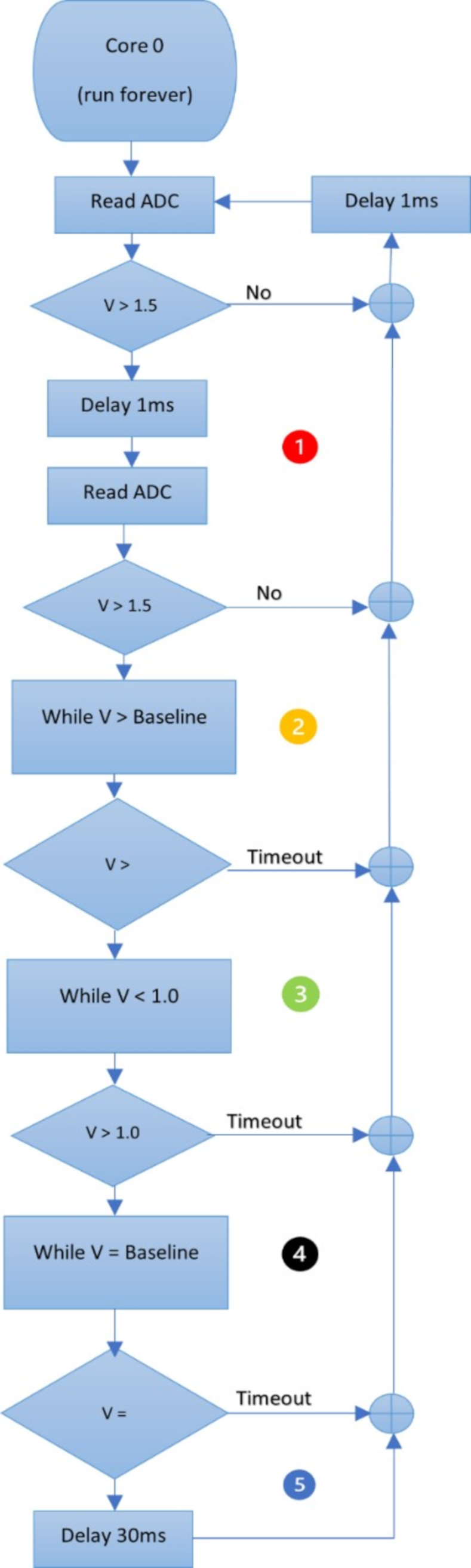

Supplement: S1 Fig — (TIF) [file pone.0290319.s001.tif]

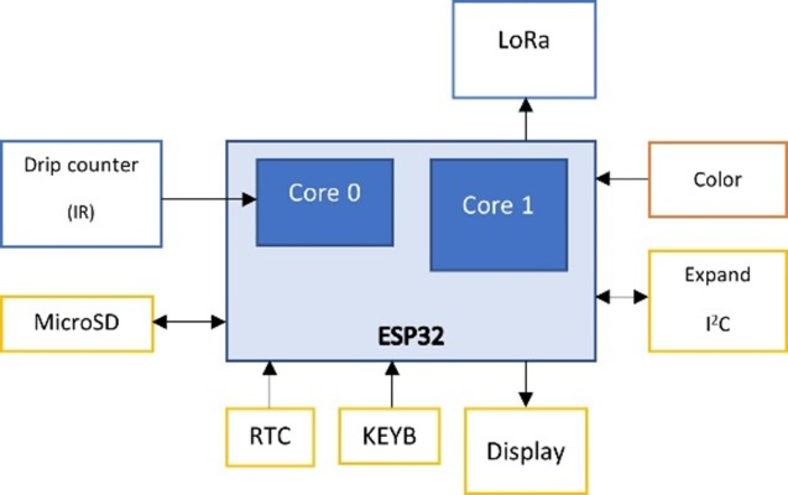

Supplement: S2 Fig — (TIF) [file pone.0290319.s002.tif]

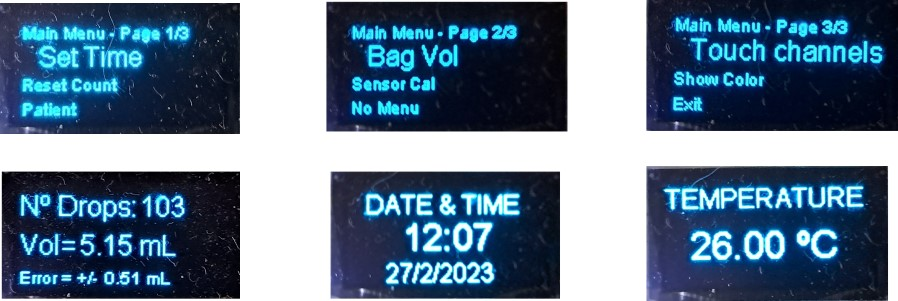

Supplement: S3 Fig — (TIF) [file pone.0290319.s003.tif]

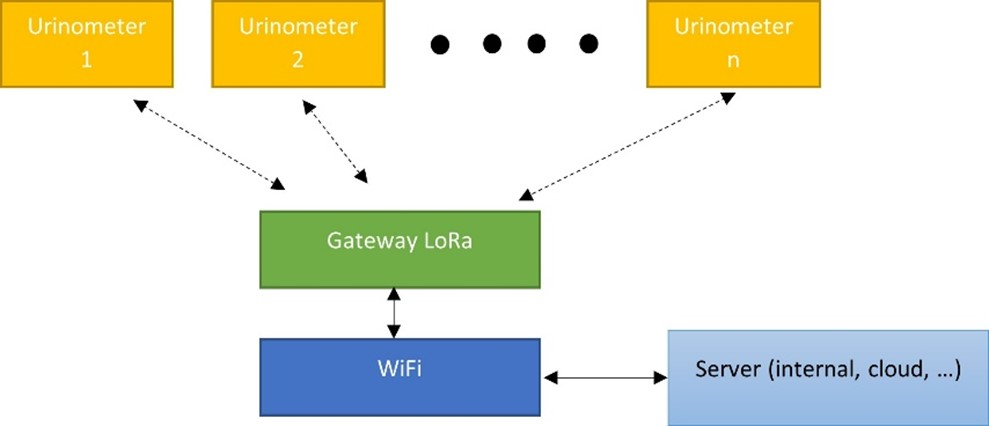

Supplement: S4 Fig — (TIF) [file pone.0290319.s004.tif]

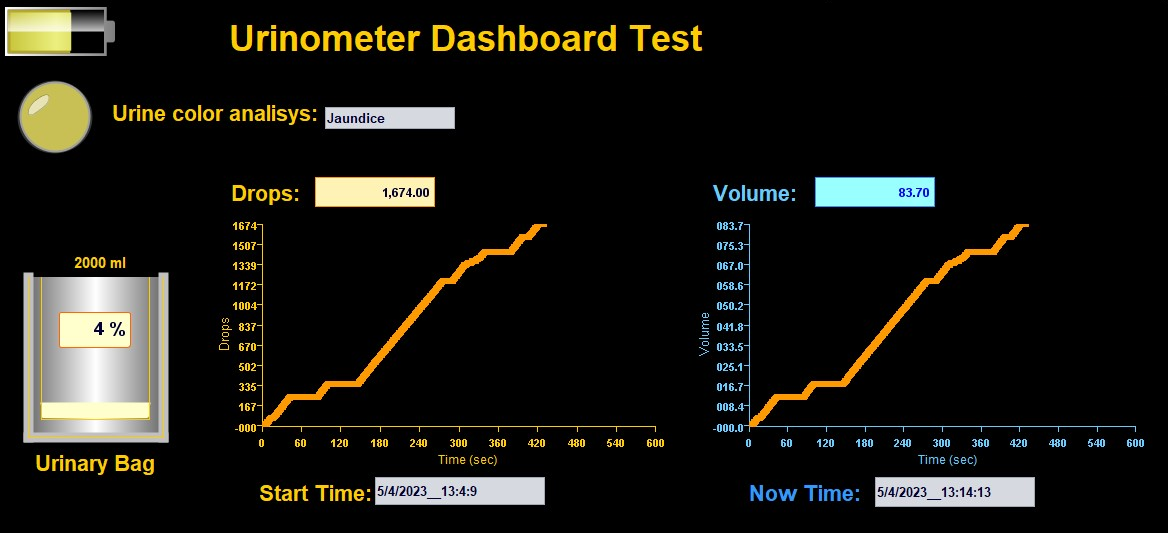

Supplement: S5 Fig — (TIF) [file pone.0290319.s005.tif]

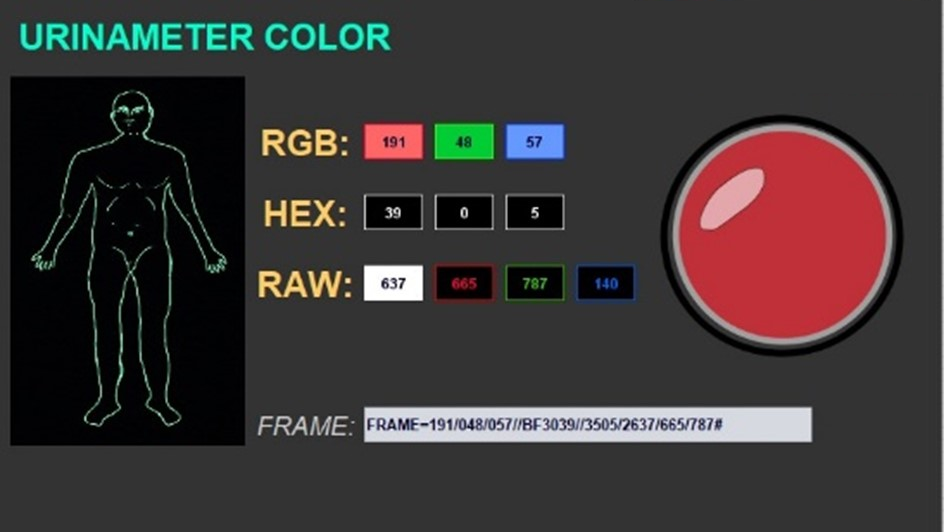

Supplement: S6 Fig — (TIF) [file pone.0290319.s006.tif]
