# Supplementary material for: Development of a urinometer for automatic measurement of urine flow in catheterized patients
Source: PLoS One. 2023 Aug 31;18(8):e0290319. doi: 10.1371/journal.pone.0290319 (PMC10470914; doi:10.1371/journal.pone.0290319)
Supplement: S1 Table — (PDF) [file pone.0290319.s007.pdf]

Urinometer Bill of Materials

Description	Quantity	Price (€)
Min PCB Model Heltec Wifi Lora 32 (V2.1)	1	18,00
Main board for urinometer (PCB)	1	3,80
PCB up ring	1	0,60
PCB down ring for sensor	1	0,60
LED IR QEE113	1	1,00
Phottransistor QSE113	1	0,80
RJ45 connector for lic ports	2	1,00
MicroSD Module	1	2,00
RTC Module with DS3231 and CR2032 battery	1	3,29
Color sensor Module TCS34725	1	3,67
MicroSD card 8Gb	1	2,68
Power Bank battery 5000 mA/h	1	15,00
Miscellaneous (wires, adaptors, connectors headers,...)	1	7,56
	**TOTAL**	**60,00**
