# Supplementary material for: Development of a urinometer for automatic measurement of urine flow in catheterized patients
Source: PLoS One. 2023 Aug 31;18(8):e0290319. doi: 10.1371/journal.pone.0290319 (PMC10470914; doi:10.1371/journal.pone.0290319)
Supplement: S2 Table — (PDF) [file pone.0290319.s008.pdf]

Density Compared

Liquid and density (g/ml)	Volume	Measured value	% error
Nrmal saline 0,9% = 1,005	100	89,95	10,05
1,005 < WATER < 1,025	100	91,50	8,50
SEAWATER => 1,0025	100	92,1	7,90

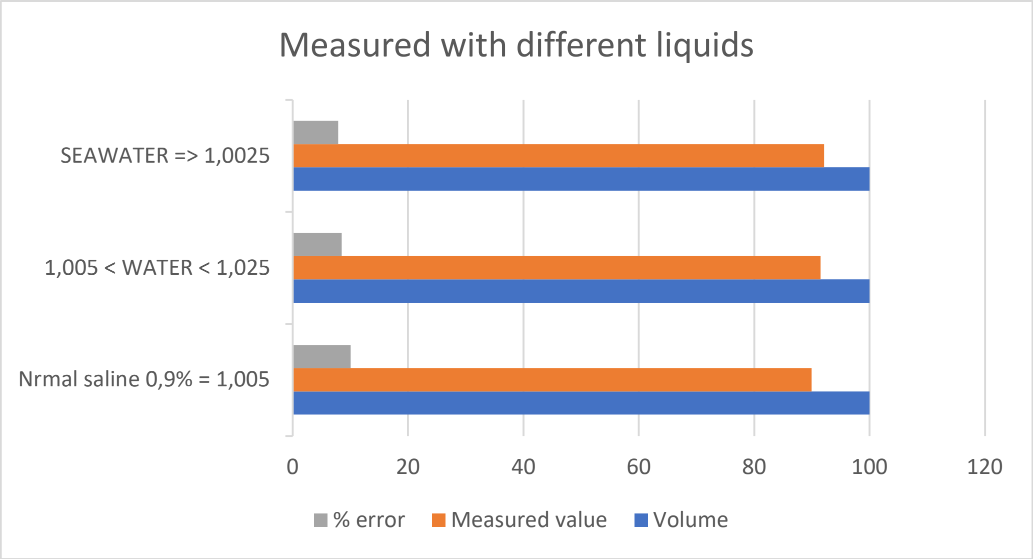

Perfusion pump			LIQUID		WATER
Flow ml/h	Volume (ml)	Measured	error %	Measurement + systomatic error	error %
10	5	4,7	6,00	5,24	4,80
10	5	4,6	8,00	5,14	2,80
10	5	4,8	4,00	5,34	6,80
10	5	4,7	6,00	5,24	4,80
10	5	4,7	6,00	5,24	4,80
10	5	4,85	3,00	5,39	7,80
10	5	4,75	5,00	5,29	5,80
50	400	363,25	9,19	406,45	1,61
50	400	364,5	8,88	407,70	1,93
50	400	362,8	9,30	406,00	1,50
50	400	362,75	9,31	405,95	1,49
50	400	363,5	9,13	406,70	1,68
50	400	363,25	9,19	406,45	1,61
60	284,12	260,75	8,23	291,43	2,57
60	284,12	259	8,84	289,68	1,96
60	284,12	262,75	7,52	293,43	3,28
60	284,12	261,25	8,05	291,93	2,75
60	284,12	260,25	8,40	290,93	2,40
60	284,12	261,25	8,05	291,93	2,75

Perfusion pump			LIQUID		WATER
Flow ml/h	Volume (ml)	Measured	error %	Measurement + systomatic error	error %
70	5	4,55	9,00	5,09	1,80
70	5	4,4	12,00	4,94	1,20
70	5	4,45	11,00	4,99	0,20
70	5	4,55	9,00	5,09	1,80
70	5	4,3	14,00	4,84	3,20
70	5	4,55	9,00	5,09	1,80
70	5	4,45	11,00	4,99	0,20
70	10	8,9	11,00	9,98	0,20
70	10	9,1	9,00	10,18	1,80
70	10	9,05	9,50	10,13	1,30
70	10	9,1	9,00	10,18	1,80
70	10	9,05	9,50	10,13	1,30
70	25	20,55	17,80	23,25	7,00
70	25	20,65	17,40	23,35	6,60
70	30	26,8	10,67	30,04	0,13
70	30	26,55	11,50	29,79	0,70
70	50	48,25	3,50	53,65	7,30
70	50	45,45	9,10	50,85	1,70
70	50	48,40	3,20	53,80	7,60
70	50	44,5	11,00	49,90	0,20
70	70	62,6	10,57	70,16	0,23

Perfusion pump			LIQUID		WATER
Flow ml/h	Volume (ml)	Measured	error %	Measurement + systomatic error	error %
70	100	84,7	15,30	95,50	4,50
70	100	90,2	9,80	101,00	1,00
70	100	90,25	9,75	101,05	1,05
70	100	89,50	10,50	100,30	0,30
70	100	88,75	11,25	99,55	0,45
70	100	89,75	10,25	100,55	0,55
70	100	90,50	9,50	101,30	1,30
70	100	92,50	7,50	103,30	3,30
70	100	94,25	5,75	105,05	5,05
70	100	95,60	4,40	106,40	6,40
70	100	95,90	4,10	106,70	6,70
70	100	91,50	8,50	102,30	2,30
70	100	94,10	5,90	104,90	4,90
70	100	93,45	6,55	104,25	4,25
70	100	91,75	8,25	102,55	2,55
70	100	92,05	7,95	102,85	2,85
70	100	92,10	7,90	102,90	2,90
70	100	92,95	7,05	103,75	3,75
70	100	95,95	4,05	106,75	6,75
70	100	92,30	7,70	103,10	3,10
70	100	96,00	4,00	106,80	6,80

Perfusion pump			LIQUID		WATER
Flow ml/h	Volume (ml)	Measured	error %	Measurement + systomatic error	error %
100	5	4,5	10,00	5,04	0,80
100	5	4,45	11,00	4,99	0,20
100	5	4,5	10,00	5,04	0,80
100	5	4,7	6,00	5,24	4,80
100	100	91	9,00	101,80	1,80
100	100	91,4	8,60	102,20	2,20
100	100	88,85	11,15	99,65	0,35
100	100	85,4	14,60	96,20	3,80
100	100	79,05	20,95	89,85	10,15
100	100	85,7	14,30	96,50	3,50
100	100	79,55	20,45	90,35	9,65
100	100	78,25	21,75	89,05	10,95
100	100	90,3	9,70	101,10	1,10
100	100	90,55	9,45	101,35	1,35
150	50	41,05	17,90	46,45	7,10
150	50	41,5	17,00	46,90	6,20
150	50	41,75	16,50	47,15	5,70
150	50	40,95	18,10	46,35	7,30
150	50	41	18,00	46,40	7,20
150	50	41,05	17,90	46,45	7,10
150	50	41,75	16,50	47,15	5,70

Perfusion pump			LIQUID		WATER
Flow ml/h	Volume (ml)	Measured	error %	Measurement + systomatic error	error %
200	50	41,05	17,90	46,45	7,10
200	100	90,15	9,85	100,95	0,95
200	100	83,75	16,25	94,55	5,45
200	100	82,5	17,50	93,30	6,70
200	100	82,75	17,25	93,55	6,45
200	100	81,95	18,05	92,75	7,25
200	100	83,25	16,75	94,05	5,95
200	100	83,5	16,50	94,30	5,70
200	100	83,9	16,10	94,70	5,30
200	200	162,6	18,70	184,20	7,90
300	100	79,55	20,45	90,35	9,65
300	100	80,05	19,95	90,85	9,15
500	16	12	25,00	13,73	14,20
500	16	11,85	25,94	13,58	15,14
500	16	12,2	23,75	13,93	12,95
500	100	77,2	22,80	88,00	12,00
500	100	76,95	23,05	87,75	12,25
500	100	76,8	23,20	87,60	12,40
500	100	76,75	23,25	87,55	12,45

Perfusion pump			LIQUID		Serum 0,9%
Flow ml/h	Volume (ml)	Measured	error %	Measurement + systomatic error	error %
100	100	89,9	10,10	100,70	0,70
100	100	89,8	10,20	100,60	0,60
100	100	89,9	10,10	100,70	0,70
100	100	89,25	10,75	100,05	0,05
100	100	89,25	10,75	100,05	0,05
100	100	89,95	10,05	100,75	0,75
100	100	89,85	10,15	100,65	0,65
100	100	89,25	10,75	100,05	0,05
100	100	89,2	10,80	100,00	0,00
100	100	89,85	10,15	100,65	0,65
100	100	89,8	10,20	100,60	0,60
100	100	89,95	10,05	100,75	0,75
100	100	89,25	10,75	100,05	0,05
100	100	89,85	10,15	100,65	0,65
100	100	89,9	10,10	100,70	0,70
100	100	89,85	10,15	100,65	0,65
100	100	89,8	10,20	100,60	0,60
100	100	89,25	10,75	100,05	0,05
100	100	89,9	10,10	100,70	0,70
100	100	89,85	10,15	100,65	0,65

Perfusion pump			LIQUID		Serum 0,9%
Flow ml/h	Volume (ml)	Measured	error %	Measurement + systomatic error	error %
100	100	89,25	10,75	100,05	0,05
100	100	89,95	10,05	100,75	0,75
100	100	89,9	10,10	100,70	0,70
100	100	89,85	10,15	100,65	0,65
100	100	89,8	10,20	100,60	0,60
100	100	89,85	10,15	100,65	0,65
100	100	89,95	10,05	100,75	0,75
100	100	89,85	10,15	100,65	0,65
100	100	89,8	10,20	100,60	0,60
100	100	89,85	10,15	100,65	0,65
100	100	89,85	10,15	100,65	0,65
100	100	89,9	10,10	100,70	0,70
100	100	89,25	10,75	100,05	0,05
100	100	89,95	10,05	100,75	0,75
100	100	89,25	10,75	100,05	0,05
100	100	89,85	10,15	100,65	0,65
100	100	89,9	10,10	100,70	0,70
100	100	89,8	10,20	100,60	0,60
100	100	89,85	10,15	100,65	0,65
100	100	89,9	10,10	100,70	0,70

Perfusion pump			LIQUID		Serum 0,9%
Flow ml/h	Volume (ml)	Measured	error %	Measurement + systomatic error	error %
200	100	80,65	19,35	91,45	8,55
200	100	80,3	19,7	91,10	8,90
200	100	80,45	19,55	91,25	8,75
200	100	80,35	19,65	91,15	8,85
200	100	80,55	19,45	91,35	8,65
200	100	80,3	19,7	91,10	8,90
200	100	80,4	19,6	91,20	8,80
200	100	80,55	19,45	91,35	8,65
200	100	80,6	19,4	91,40	8,60
300	100	79,85	20,15	90,65	9,35
300	100	79,9	20,10	90,70	9,30
300	100	79,85	20,15	90,65	9,35
300	100	80,15	19,85	90,95	9,05
350	100	78,95	21,05	89,75	10,25
400	100	78,1	21,90	88,90	11,10
400	100	77,6	22,40	88,40	11,60
500	100	79,2	20,80	90,00	10,00
500	100	79	21,00	89,80	10,20
500	100	79,15	20,85	89,95	10,05
500	100	78,95	21,05	89,75	10,25

Perfusion pump			LIQUID	AGUA	Sea water
Flow ml/h	Volume (ml)	Measured	error %	Measurement + systomatic error	error %
100	100	90,85	9,15	101,65	1,65
100	100	90,95	9,05	101,75	1,75
100	100	89,9	10,10	100,70	0,70
100	100	91,95	8,05	102,75	2,75
100	100	90,85	9,15	101,65	1,65
100	100	91,95	8,05	102,75	2,75
100	100	90,95	9,05	101,75	1,75
100	100	89,25	10,75	100,05	0,05
100	100	91,95	8,05	102,75	2,75
100	100	90,85	9,15	101,65	1,65
100	100	89,8	10,20	100,60	0,60
100	100	90,95	9,05	101,75	1,75
100	100	89,25	10,75	100,05	0,05
100	100	90,85	9,15	101,65	1,65
100	100	90,85	9,15	101,65	1,65
100	100	89,85	10,15	100,65	0,65
100	100	91,95	8,05	102,75	2,75
100	100	89,25	10,75	100,05	0,05
100	100	91,95	8,05	102,75	2,75
100	100	90,85	9,15	101,65	1,65

Nº	COLOR	SAMPLE COLOR	R	G	B	CLEAR	TEMP	LUX
1		NO TUBE	185	255	255	65535	11176	40026
2		EMPTY TUBE	124	117	86	65535	4127	21380
3		TUBE WITH WATER	128	146	110	65535	5065	27962
4		LIGHT YELLOW	99	113	65	48694	3867	18802
5		STRAW YELLOW	113	89	67	45008	3454	9559
6		GREENISH-YELLOW	94	105	69	49674	4268	16571
7		YELLOW VERY CLEAR	101	99	75	40541	4519	10995
8		CLEAR YELLOW	105	92	70	49977	4008	11808
9		ORANGE/YELLOW	135	90	39	49092	2129	13402
10		ORANGEGOLD	154	77	54	32191	1654	4309
11		PINK	183	55	54	19435	6908	64587
12		RED/ORANGE	160	69	70	26746	3933	806
13		RED WINE	164	68	70	25584	5647	367
14		RED WINE	129	83	77	18253	3638	2770
15		DARK RED	134	95	92	15874	4669	2503
16		LIGHT GREEN	90	105	85	32389	5704	9541
