# Supplementary material for: Development of a urinometer for automatic measurement of urine flow in catheterized patients
Source: PLoS One. 2023 Aug 31;18(8):e0290319. doi: 10.1371/journal.pone.0290319 (PMC10470914; doi:10.1371/journal.pone.0290319)
Supplement: S1 Appendix — (PDF) [file pone.0290319.s009.pdf]

S1 Appendix. Physiological and clinical data.

To provide further clarification and address clinical or medical inquiries pertaining to the urinary system and its impact on urinometer readings, we have attached a document that answers common questions in this field of study.

There may be instances where urine flow rates are unclear, leading to doubts about the ability of the urinometer to accurately measure these flows. In order to address this, we have provided a detailed explanation of the urinary system and its normal functioning (as studied in physiology), as well as its functioning when catheterized with a Foley probe, which can cause changes in certain aspects of the urinary system's function.

Our proposed urinometer operates on the principle of gravity. It should be noted that the flow of urine through the ureters and subsequently through the urethra (as depicted in the image below) is typically less than the flow admitted by the dropper.

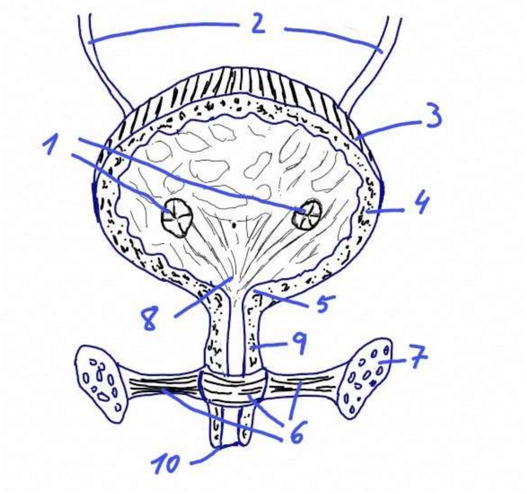

1. Ureteral openings
2. Ureters
3. Bladder
4. Detrusor muscle
5. Internal urethral sphincter (involuntary)
6. External urethral sphincter (voluntary)
7. Hip bone (pubis)
8. Internal urethral orifice
9. Urethra
10. External urethral orifice

Fig. 1. Physiology of the bladder. Source: authors.

In our study, we rely on the expertise of our clinical staff who have tested the dropper with 0.9% saline solution and have determined that it can admit up to 1200mL per hour, which amounts to 28.8 liters per day. This level of fluid intake is considered pathological and indicates polydipsia. Based on this information, we conclude that, under normal physiological and pathophysiological conditions (excluding rare ureteral and/or urethral pathologies), the dropper is unlikely to pose a problem for the patient's urine flow.

When a patient is catheterized, the flow of urine typically occurs by gravity, and thus, the urine bag is placed below the patient's bladder to prevent retrograde flow. While the stream of urine depends on the muscular contraction of the bladder wall under normal conditions, with a catheter, an accumulation of fluid can occur in the bladder that can be released instantly and cause a jet flow by free fall. Nonetheless, we know that the flow of urine will mostly be constant and lower than the flow admitted by the dropper, based on our understanding of human physiology.

Our system aims to detect the jet flow of urine with a sensor that utilizes pattern recognition to capture the volume through recognizable patterns, thereby minimizing measurement error. This approach is different from other devices that do not measure anything during the jet event. To provide a better understanding of this topic, we reviewed renal physiology without and with a Foley probe, relying on the works of Silverthorn and Derrickson.

In healthy individuals, urine forms in the kidneys and is transported to the bladder through the ureters via peristaltic contractions. The detrusor muscle in the bladder wall can hold around 700-800mL of urine on average. Urination is caused by a combination of involuntary and voluntary muscle contractions triggered by the stretching receptors of the bladder wall, which transmit signals to the urination center in the spinal cord. The spinal reflex causes the detrusor muscle to contract and the internal urethral sphincter to relax, allowing urine to flow out.

However, when a patient is catheterized, the system changes, and the catheter inhibits the functioning of both internal and external sphincters, turning the system into a gravity system. The urine bag must always be placed below the bladder to prevent retrograde flow. With this understanding of renal physiology, we can conclude that our sensor system is designed to detect the jet flow of urine accurately and effectively.

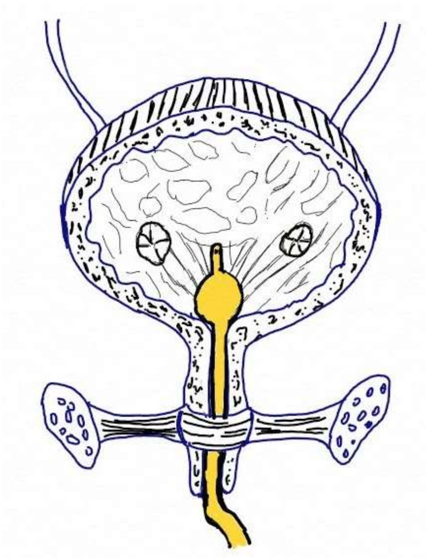

Fig. 2. In yellow Foley probe or urinary catheter, blocking (inhibiting) the functions of the sphincters. Source: authors.

The phenomenon known as the jet effect can occur when a patient with a urinary catheter assumes different positions, causing the bladder to accumulate some urine that does not reach the level of the catheter's exit hole. The occurrence of this effect depends on the patient's posture and how the catheter is positioned within the bladder. It is important to note that the jet effect is not caused by contractions of the bladder muscles or pressure from the detrusor muscle, but rather by the patient's movements and resulting mechanical changes in the bladder.

In some cases, the catheter may become obstructed by the fully contracted bladder, leading to urine accumulation and bladder expansion, which in turn causes the patient to consciously activate the detrusor muscle. However, this pressure is highly variable and should not affect the ability of the catheter to allow urine flow. If such an occurrence were to happen, it would be isolated and infrequent. Based on our observations, we believe that urine flow is mostly constant and lower than the flow rate allowed by the catheter.

Another concern is the possibility of retrograde contamination. The device we use to measure urine output is a medical-grade dropper typically used for intravenous lines, and therefore sterile. Our electronic device is placed around the dropper, not in contact with the urine, and has no effect on the properties of the urine, including bacterial infections. Additionally, our device is situated between the Foley catheter and the urine bag, and the urine sample extraction port is located in the urine bag tube. This means that the extraction of the sample will not affect the measurement of urine volume. However, bacterial growth may still occur in the previously contaminated urine drip device, even without our urinometer.

To address this concern, we have included an image of the extraction port beneath the dropper to ensure proper placement and reduce the risk of contamination.

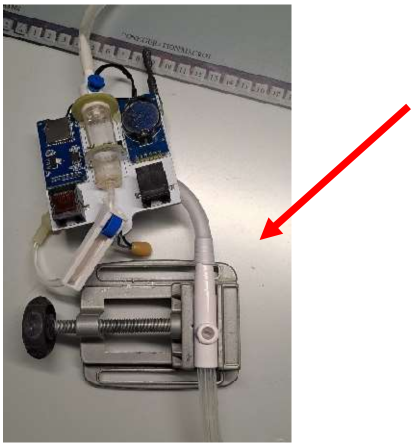

Fig. 3 Urine sample extraction port
