# Supplementary material for: Development of a urinometer for automatic measurement of urine flow in catheterized patients
Source: PLoS One. 2023 Aug 31;18(8):e0290319. doi: 10.1371/journal.pone.0290319 (PMC10470914; doi:10.1371/journal.pone.0290319)
Supplement: S2 Appendix — (PDF) [file pone.0290319.s010.pdf]

S2 Appendix. Jet detection

To detect the stream, a flow rate per unit of time should be calculated. With a continuous flow, the time elapsing from the beginning of the continuous flow until the end of the event is counted and the volume of liquid is calculated, taking the nominal flow of the dripper as a constant. The term “flow” means the volume of liquid that runs across a surface in a given time. Flow is calculated using the following formula:

$$Q = \frac{V}{t} \qquad (1)$$

where Q is the flow rate, V is the volume in milliliters, and t is the time in milliseconds. Assuming a fixed value for Q, the following formula is applied:

$$V = Q \cdot t \qquad (2)$$

once the time from the beginning of the continuous flow to the end of said flow has elapsed. The continuous flow pattern can be differentiated from the droplet pattern and its calculated flow rate.

Figure 1 shows a waveform corresponding to a constant flow pattern or continuous flow. When a first amplitude threshold is exceeded, there is a first timeout interval of 5 ms. If at this point the voltage stays above the threshold, the system waits until the voltage stabilizes at a "Baseline" level, which is the normal voltage when the liquid is not passing through. The firmware is able to capture the event and counts the time elapsed from the beginning (first threshold) to the end (second threshold) and a volume is obtained by applying the formula for continuous flow (3).

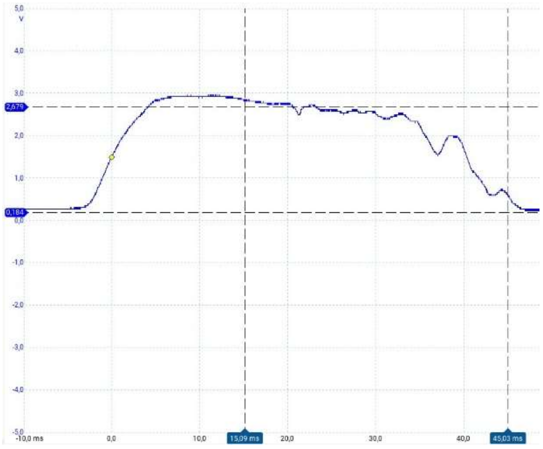

Fig. 1 Jet shape in dripper

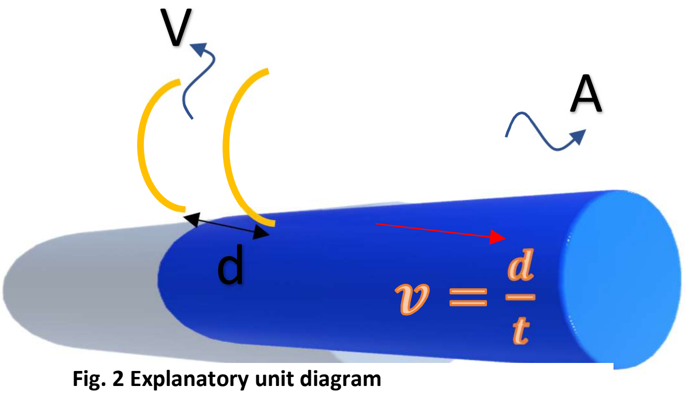

Fig. 2 Explanatory unit diagram

Knowing the time and flow capacity we can obtain the volume. according to the basic theory of fluid physics, we can have a calculation of Q (volumetric flow rate) using an alternative that will help us in our purpose. Figure 2 shows a portion of generic pipe for better understanding of the variables and their relationship.

Writing the volume of a portion of fluid in a pipe as

$V = A * d$ (3)

Being A the cross section of the fluid and d the width (length of the pipe), we substitute this formula into the general formula (1) obtaining:

$Q = \frac{V}{t} = \frac{A*d}{t} = A * \frac{d}{t}$ (4)

$\frac{d}{t}$ It refers to the length of a pipe ratio divided by the elapsed time (speed). And we get

$$Q = A * v$$

Now, everything is reduced to knowing a cross area and a distance to be able to obtain the velocity and hence the flow or flow.

Entonces definamos una porción de tubo de nuestro sistema, el gotero. Si analizamos nuestro gotero, podemos definir fácilmente una distancia d y un área. En la figura 3 podemos ver medidas aproximadas para poder incluir en los cálculos.

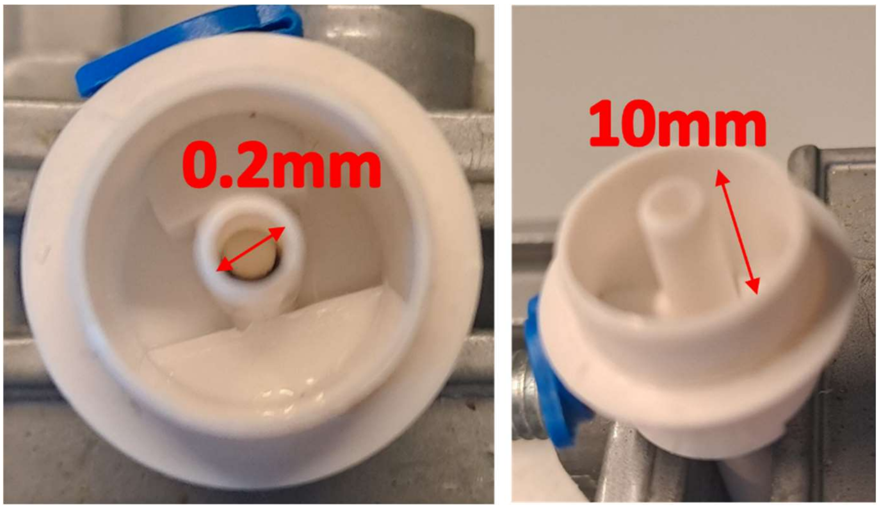

Fig. 3 Internal measures of the dropper
