# Supplementary material for: Development of a urinometer for automatic measurement of urine flow in catheterized patients
Source: PLoS One. 2023 Aug 31;18(8):e0290319. doi: 10.1371/journal.pone.0290319 (PMC10470914; doi:10.1371/journal.pone.0290319)
Supplement: S3 Appendix — (PDF) [file pone.0290319.s011.pdf]

S3 appendix. Realistic simulation model.

Preparation of a realistic simulation model.

To conduct tests on the device, it is necessary to create a simulator of the human urinary system that closely resembles the real thing (refer to Fig. 1). Based on the knowledge of the physiology of the urinary system, we developed a model using a renal simulator - a vessel equipped with a flow regulator that allows us to control the urine flow rate per hour that we want to simulate. This renal simulator feeds into a bladder made of nitrile glove, which has a Foley catheter (18 Fr in our case) inserted. To ensure accuracy and consistency in the measurements, we use an ICU urinometer (Ureofix® 500 model) for comparison. However, during the actual usage, a standard urinary bag will be used instead, as it is more cost-effective.

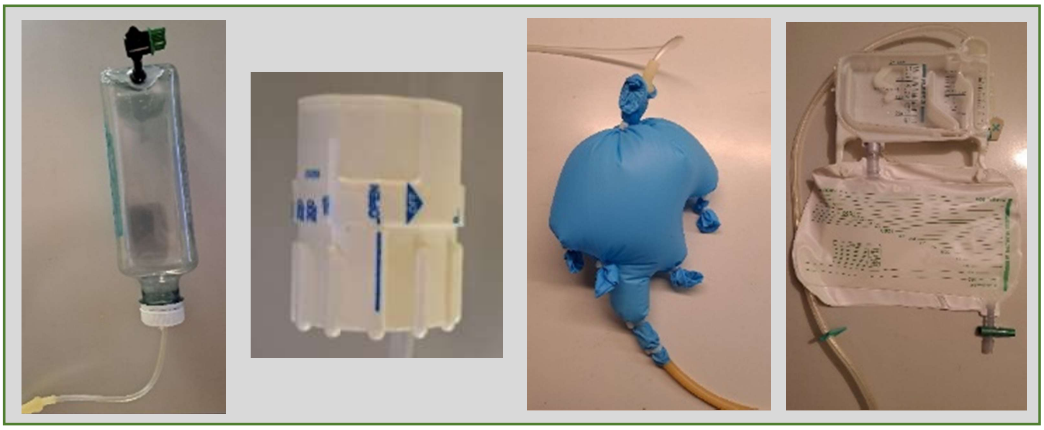

Fig 1. Elements of the renal system and urine excretion simulator. From left to right: bottle that simulates the kidneys, the flow regulator, model of the bladder with the Foley catheter and, on the right, the Ureofix 500 urinometer.

Furthermore, we conducted a test to measure the fluid flow rates through the dropper system in the Intensive Care Unit (ICU) of the HLC Moncloa hospital in Madrid, Spain. During this test, we used a B.Braun precision peristaltic pump (refer to Figure 2) which is commonly used for administering medication and food. Specifically, we tested the circulation of 0.9% physiological serum through the dropper system. Our results indicate that the liquid flows at the accuracy level stated by the manufacturer, which is within a range of +-10%. It should be noted that this error represents the maximum potential discrepancy and could be influenced by factors such as liquid remaining in the tube. Additionally, it is important to highlight that this particular infusion pump model has an error rate of +-5% as indicated in the operating manual.

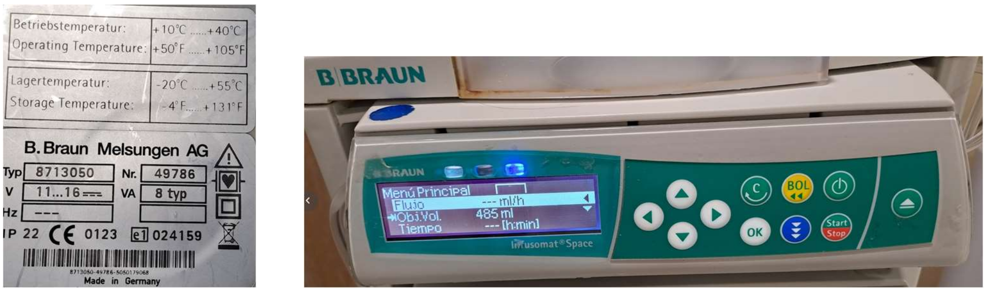

Fig 2. Braun pump used in the tests.

In our initial test, we set the pump to a flow rate of 200ml/h and observed that after 60 minutes, our device recorded a volume of 186.80 ml, resulting in an error of 6.6%. We established a criterion that our urinometer would be deemed valid if the deviation is less than 20%, as this would provide clinically relevant data for decision-making purposes.

For our second test, we conducted flow rate variations every 5 minutes to evaluate the device's performance at different flow rates within a 5-minute interval

Presetin pump (ml/h)	Flow in ml/min	Theoretical volume (ml) / 5 min	Measured volume (ml) / 5 min	Error %
450	7,50	37,50	31,75	15,33
400	6,66	33,30	29,70	10,81
350	5,83	29,16	26,05	10,67
300	5,00	25,00	23,47	6,12
250	4,16	20,80	19,50	6,25
200	3,33	16,65	15,30	8,11
150	2,50	12,50	12,50	0,00
100	1,66	8,33	6,85	17,77
80	1,33	6,65	7,05	6,02
50	0,83	4,15	3,75	9,64
30	0,50	2,50	2,75	10,00
20	0,33	1,65	1,90	15,15
10	0,17	0,83	0,80	3,61
5	0,08	0,42	0,42	0,00

In a third test these are the data collected.

Theoretical volume (ml)	Measured volume (ml)	Error %
100	104,75	4,75
100	103,35	3,35
100	101,6	1,6
100	101,6	1,6
100	98,7	1,3
100	97,75	2,25
100	96,15	3,85
100	95,6	4,4
100	99,1	0,9
100	103,95	3,95
100	94,1	5,9
100	93,45	6,55
100	91,75	8,25
100	92,05	7,95
100	92,1	7,9
100	92,95	7,05
100	95,95	4,05
100	92,3	7,7
100	97	3
50	51,4	2,8
50	45,45	9,1
50	48,4	3,2

Improved pattern detection has led to better capture results. Jet detection is detected easily in the initial stages. In the future, we will be able to evaluate its calculation by determining the maximum and minimum flow allowed by the dropper and the elapsed time. We were unable to physically compare our device with other equipment because no commercial device is available in Spain. However, we have evidence that experimental tests have been conducted in some hospitals in our vicinity. In addition, the comparisons we make with scientific publications and information available on web pages are limited due to insufficient information.
